# Design Rules for Bimetallic and Multimetallic Nanocatalysts Harnessing Synergistic Effects for Alkaline Hydrogen Oxidation

**DOI:** 10.1021/acsnanoscienceau.6c00055

**Published:** 2026-07-06

**Authors:** Yun-Shan Tsai, Shang-Cheng Lin, Cheng-Yu Wu, Tung-Han Yang

**Affiliations:** † Department of Chemical Engineering, 34881 National Tsing Hua University, Hsinchu 300044, Taiwan; ‡ High Entropy Materials Center, National Tsing Hua University, Hsinchu 300044, Taiwan; § College of Semiconductor Research, National Tsing Hua University, Hsinchu 300044, Taiwan

**Keywords:** alkaline hydrogen oxidation reaction, electrocatalysts, bimetallic catalysts, multimetallic catalysts, high-entropy alloy catalysts, density functional theory, hydrogen binding energy, hydroxyl binding energy, interfacial water

## Abstract

Anion-exchange membrane fuel cells offer a promising route for hydrogen-to-electricity conversion under alkaline conditions, yet the anode hydrogen oxidation reaction (HOR) remains kinetically sluggish. This Review summarizes key mechanistic views of alkaline HOR, including hydrogen binding, hydroxyl binding, bifunctional effects, and emerging roles of interfacial water and solvation. We highlight experimental tools, such as electrochemical adsorption probes and operando vibrational and X-ray spectroscopies, that reveal potential-dependent surface states and interfacial species. We also discuss theoretical approaches, including density functional theory and data-driven models, for evaluating ensemble effects and elementary reaction barriers. Building on these insights, we outline catalyst design principles for bimetallic and multimetallic nanocatalysts, including high-entropy alloys, emphasizing adsorption-strength tuning, ensemble control, and poisoning tolerance. These discussions provide a basis for rational catalyst optimization under practical alkaline fuel-cell conditions. Finally, we propose six future directions to bridge mechanistic understanding and practical catalyst development for durable, device-relevant alkaline HOR catalysts.

## Graphical abstract

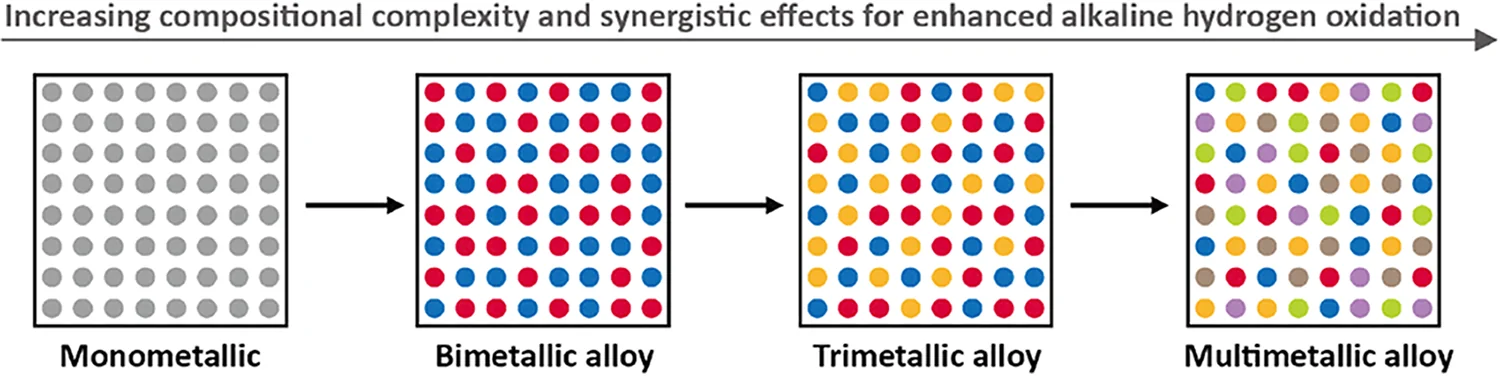

## 1 Introduction

Anion exchange membrane fuel cells (AEMFCs) continue to attract attention as a practical route to efficient hydrogen-to-electricity conversion in alkaline environments.^1–5^ By operating under alkaline conditions, AEMFCs can, in principle, expand the set of stable catalysts and supports and reduce precious-metal dependence, while still pairing hydrogen oxidation reaction (HOR) at the anode with oxygen reduction reaction (ORR) at the cathode.^6–8^ Yet, even with rapid progress in membranes, ionomers, and electrode architectures, the anode HOR remains a persistent kinetic bottleneck. Relative to acidic electrolytes, HOR on benchmark noble metals is substantially slower in alkaline media, raising the required catalyst loading and narrowing the performance margin in real devices.^9–11^ Closing this alkaline kinetic gap is therefore both a fundamental problem in electrocatalysis and a practical materials challenge for enabling durable, high-power AEMFCs. In alkaline electrolytes, HOR is commonly framed in terms of a few elementary motifs that couple hydrogen activation to an interfacial charge-transfer step that regenerates surface sites while producing water. This framework is useful, but it does not fully resolve the design-relevant questions. Specifically, it remains unclear which motif controls the rate within a given potential window and how surface coverages evolve under polarization. It also does not explain why HOR kinetics can depend so strongly on electrolyte composition and double-layer structure. The core challenge is that, under alkaline conditions, hydrogen chemistry cannot be cleanly separated from hydroxide and water chemistry at the interface. Adsorbed hydrogen must form and be removed efficiently, yet its removal is tied to how hydroxide is delivered to, and reorganized within, the interfacial region, either through solvated hydroxide participating directly or through oxygenated adsorbates stabilized on the surface. As a result, mechanistic interpretations that look straightforward in acid often become conditional in alkaline media, depending on potential, surface state, and the local interfacial environment.

Descriptor-based thinking has therefore played a central role, particularly the hydrogen binding energy (HBE). The premise is simple: overly weak H* binding penalizes hydrogen activation, whereas overly strong binding hinders site regeneration and charge transfer.^12–16^ HBE remains attractive because it provides a thermodynamic handle that can rationalize activity trends across metals and alloys. Still, alkaline HOR rarely reduces to a single descriptor. In many cases, catalysts that bring HBE toward an “optimal” value do not deliver commensurate gains unless hydroxide-related processes are also favorable. This motivates the hydroxyl binding energy (OHBE) and bifunctional perspectives, where oxophilic sites promote the formation and accommodation of oxygenated intermediates that couple with H* to form water.^17,18^ The debate is not whether HBE or OHBE matters, but when each one dominates, how they interact under realistic coverages, and whether they can be treated independently. In practice, H* and OH* can compete for surface ensembles, and their coverages may shift rapidly with potential and electrolyte conditions. These coupled effects complicate attempts to identify a single rate-limiting step or to interpret kinetics using equilibrium adsorption energetics defined under idealized conditions.

More recently, interfacial water has moved from a background consideration to a central mechanistic variable. In alkaline HOR, water is not only a product; it is also a structural element of the double layer and an active participant that governs hydroxide approach, partial desolvation, and transition-state stabilization through hydrogen-bond networks.^19,20^ Cation identity and concentration can reorganize this interfacial water structure, reshape local electric fields, and shift effective barriers associated with hydroxide transfer and interfacial reorganization.^21^ These effects can change kinetics even on nominally identical surfaces, providing a natural explanation for trends that cannot be captured by HBE or OHBE alone. From this viewpoint, the relevant kinetic barrier often includes solvent-reorganization and interfacial-transport contributions in addition to the intrinsic surface chemistry of H* and OH*.^22,23^ This becomes particularly important when comparing catalysts with different oxophilicity or hydrophilicity, since those properties can reshape local water structure and influence both hydroxide accessibility and poisoning susceptibility.^24,25^ Interfacial water therefore needs to be considered alongside H* and OH* energetics when formulating design rules for alkaline HOR.

This mechanistic picture also helps explain why alloying and multimetallic design have been so effective. Bimetallic and multimetallic catalysts offer a way to separate requirements that are difficult to satisfy on a single metal.^18,26–29^ A surface optimized for hydrogen activation is not necessarily optimal for hydroxide handling, and a surface that readily stabilizes oxygenated species can suffer from site blocking.^30^ The most compelling designs therefore create complementary motifs on the same catalyst. Ensembles that favor hydrogen activation are positioned near ensembles that facilitate hydroxyl-related steps and water formation. As a result, the overall reaction can proceed through cooperative pathways with reduced competition for adsorption. This leads to three recurring design targets. The surface should offer distinct site types for hydrogen- and hydroxyl-related chemistry, distribute these motifs so that they can act cooperatively at the nanometer scale, and maintain resistance to poisoning and potential-driven reconstruction. In practice, these targets can impose trade-offs, for example, increasing oxophilicity to promote hydroxyl chemistry may also raise oxygenated-species coverage, underscoring the need for mechanism-informed design rules. The apparent contradictions across the literature often reflect the fact that internal variables (composition, atomic arrangement, coordination heterogeneity) are entangled with external variables (electrolyte identity and local pH environment). Disentangling these couplings is essential for moving beyond empirical alloy screening.

Here, we focus on design rules for bimetallic and multimetallic nanocatalysts that harness synergistic effects to overcome alkaline HOR limitations. We first summarize the major mechanistic frameworks and their points of contention, emphasizing how HBE, OHBE, and interfacial water can each dominate, or cogovern, alkaline HOR kinetics. We then outline experimental and computational strategies to disentangle these coupled effects. Experimentally, electrochemical measurements such as hydrogen underpotential deposition (HUPD) and CO-stripping provide quantitative readouts of hydrogen adsorption behavior, accessible surface ensembles and electrochemically active surface area, as well as surface cleanliness and poisoning tolerance.^11,23,31–33^ Mechanistic spectroscopies, including operando vibrational and X-ray-based methods, can probe potential-dependent surface states and interfacial species, linking measured kinetics to the evolving populations of adsorbates and the local solvent structure.^34–39^ On the modeling side, density functional theory (DFT) can be used to evaluate adsorption energetics and elementary reaction barriers. DFT also provides a quantitative framework to interpret interelement interactions and synergistic effects in bimetallic and multimetallic ensembles, including those arising from changes in local coordination environments.^37,39,40^

Building on this mechanistic foundation, we survey representative catalyst families from bimetallic systems, including noble–noble and noble-non-noble combinations, to trimetallic catalysts and high-entropy alloys (HEAs). Across these categories, we emphasize how synergy can be engineered through elemental selection to tune electronic structure, ensemble availability, and adsorption-strength distributions, thereby addressing the coupled requirements of hydrogen activation, hydroxyl management, and compatibility with the interfacial-water network. Finally, we identify six critical directions that we believe will shape the next stage of alkaline HOR research toward practical AEMFC anodes. Together, these efforts can move the field from descriptive correlations to design rules that remain robust across catalysts, electrolytes, and operating environments, ultimately enabling alkaline HOR electrocatalysts that combine high activity with the long-term stability required for AEMFC deployment. To provide a visual overview of this Review, Figure 1 summarizes the scope and organization of the discussion. The scheme highlights the progression from monometallic catalysts to bimetallic, trimetallic, and multimetallic alloy catalysts, and connects this compositional complexity with the key mechanistic concepts, analytical methods, and catalyst design strategies discussed throughout the Review.

**Figure 1:**
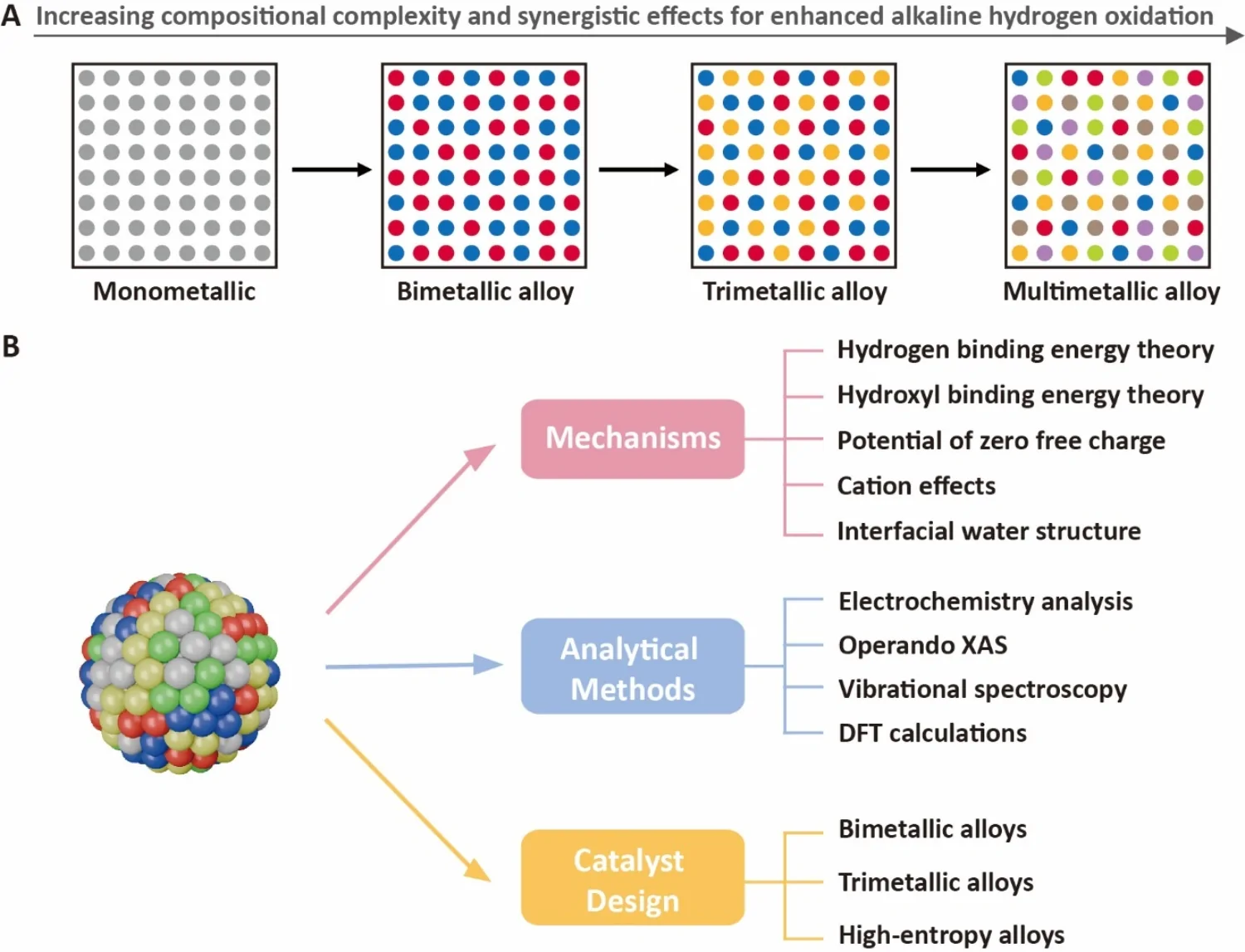
Overview of the scope and design framework of this Review. (A) Schematic illustration of the progression from monometallic catalysts to bimetallic, trimetallic, multimetallic alloy catalysts, highlighting increasing compositional complexity and synergistic effects. (B) Conceptual roadmap of the Review, connecting mechanistic understanding, characterization and modeling methods, and catalyst design strategies for alkaline HOR. The mechanistic discussion covers HBE, OHBE, potential of zero free charge effects, cation effects, and interfacial water structure. The methodological framework includes electrochemical analysis, *operando* XAS, vibrational spectroscopy, and DFT. The catalyst design section summarizes representative noble–noble, mixed noble and non-noble, non-noble, trimetallic, and HEA catalyst systems.

## 2 Alkaline HOR Mechanisms, Interfacial Effects, and Kinetic Metrics

In this section, we present an integrated mechanistic and interfacial view of alkaline HOR to connect fundamental kinetics with catalyst design and performance evaluation. We first consolidate current mechanistic understanding, highlighting plausible elementary steps and key kinetic signatures. We then discuss hydrogen binding energy as a primary adsorption descriptor that rationalizes activity trends across compositions and surface structures. In parallel, we evaluate hydroxyl binding energy and the bifunctional concept to clarify how OH-related intermediates and water activation can facilitate HOR in alkaline media. We further emphasize interfacial environment effects, including interfacial water structure, local solvation, cation-specific effects, and potential-dependent interfacial properties that modulate adsorption energetics and reaction kinetics. Finally, we outline quantitative metrics and benchmarking practices to enable consistent, reproducible comparisons of alkaline HOR catalysts across studies.

### 2.1 Mechanistic Understanding of HOR

The alkaline HOR serves as the anode half-reaction in AEMFCs, where H_2_ is oxidized to generate electrons and form water (eq 1):

1 $$\mathrm{Overallreaction}:H_{2}+2{\mathrm{OH}}^{-}\to 2H_{2}O+2e^{-}$$

Generally, alkaline HOR proceeds through three elementary steps: Tafel, Heyrovsky, and Volmer (eqs 2–4). Both the Tafel and Heyrovsky steps involve the dissociation of molecular hydrogen. In the Tafel step, H_2_ dissociates on two adjacent catalytic sites to form adsorbed hydrogen (H_ad_) without charge transfer. In contrast, the Heyrovsky step is an electrochemical dissociation step, in which H_2_ dissociation is coupled with electron transfer in the presence of OH^–^, yielding H_ad_ and water. The resulting H_ad_ is then oxidized and removed via the Volmer step by reacting with OH^–^ to form water while releasing electrons. Accordingly, alkaline HOR is commonly described by two overall pathways, namely the Tafel-Volmer and Heyrovsky-Volmer pathways.^4,6,7,41–43^

2 $$\mathrm{Tafel}:H_{2}+2M^{*}\to 2M^{*}-H_{\mathrm{ad}}$$

3 $$\mathrm{Heyrovsky}:H_{2}+{\mathrm{OH}}^{-}+M^{*}\to M^{*}-H_{\mathrm{ad}}+H_{2}O+e^{-}$$

4 $$\mathrm{Volmer}:M^{*}-H_{\mathrm{ad}}+{\mathrm{OH}}^{-}\to H_{2}O+e^{-}+M^{*}$$

Mechanistic interpretations of alkaline HOR have largely been framed around the adsorption of key intermediates, with HBE and OHBE forming two widely adopted descriptor-based viewpoints. Within the HBE framework, the interaction strength between H and surface sites is treated as a primary determinant of reaction rates and is commonly rationalized through the Sabatier principle.^9,11^ By contrast, the OHBE framework emphasizes oxophilic motifs that can facilitate the oxidative removal of H_ad_ via cooperative interactions with OH-related species and is often discussed under the umbrella of bifunctional concepts.^17,18,24,44^

Despite their utility, adsorption-centric descriptors do not consistently account for the full range of experimental observations. A particularly well-known example is the alkaline kinetic gap, namely that HOR is often systematically slower in alkaline electrolytes than in acidic environments even on benchmark Pt-based catalysts, as reflected by lower exchange current densities and larger overpotentials under comparable conditions.^9–11^ A key limitation is that HBE and OHBE predominantly capture adsorption thermodynamics on idealized surfaces, whereas alkaline HOR is strongly modulated by potential-dependent interfacial electrostatics and solvent organization, which can reshape effective adsorption energetics and activation barriers.^22,23,45^ Accordingly, complementary perspectives have been advanced that highlight interfacial and electrostatic contributions, including potential of zero free charge (*E*_pzfc_), electrolyte cation effects, and the structuring of interfacial water.^46,47^ By explicitly considering the EDL and solvent reorganization at the electrode–electrolyte interface, these approaches broaden the mechanistic narrative beyond adsorption energetics alone.^48,49^

### 2.2 Hydrogen Binding Energy (HBE) Theory

HBE serves as a foundational reaction descriptor governing the kinetics of the HOR.^50,51^ Given that H_ad_ is the primary surface intermediate in the HOR process, its binding stability on the catalyst surface directly dictates the overall reaction rate and accessible kinetic pathways.^52^ In heterogeneous catalysis, the Sabatier principle provides the theoretical cornerstone for the rational design of high-performance catalysts, stipulating that an optimal surface must exhibit a moderate adsorption strength for key reaction intermediates.^12–14^ As schematically illustrated in Figure 2, excessively weak binding limits hydrogen activation, whereas overly strong binding leads to surface poisoning and hinders the regeneration of active sites. Consequently, HOR kinetics are intrinsically governed by the adsorption–desorption dynamics of H_ad_. This relationship positions the Δ*G*_H_ as a crucial descriptor for rationalizing catalytic activity trends across diverse material systems.^14^

**Figure 2:**
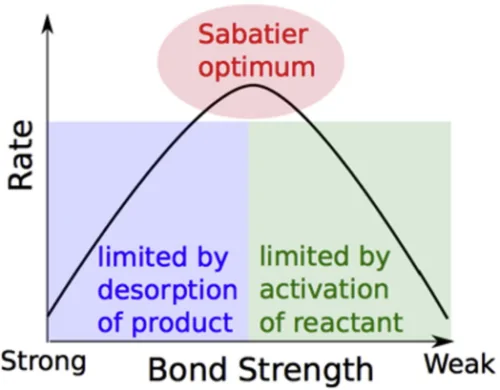
Schematic illustration of the qualitative Sabatier principle, showing the volcano-type dependence of reaction rate on bond strength.^14^ Reprinted from Journal of Catalysis, Vol. 328, Andrew J. Medford et al., “From the Sabatier principle to a predictive theory of transition-metal heterogeneous catalysis,” Pages 36–42, Copyright (2015), with permission from Elsevier.

Within the framework of DFT, HBE is commonly quantified by Δ*G*_H_, which serves as a quantitative descriptor of hydrogen adsorption on the electrode surface. When catalytic activity is correlated with Δ*G*_H_, a characteristic volcano-type relationship emerges, wherein optimal HOR activity is achieved as Δ*G*_H_ approaches thermoneutrality (Δ*G*_H_ = 0 eV). At this apex, the catalyst attains a balance between efficient hydrogen activation and rapid product release. Correlating exchange current densities (*j*_0_) with DFT-derived Δ*G*_H_ values yields characteristic volcano-type relationships. Notably, platinum-group metals (PGMs) are located near the summit of these volcano plots, reflecting their near-optimal hydrogen adsorption energetics and superior intrinsic catalytic performance.^50,51^

In 2013, Yan and co-workers first proposed a strategic approach to evaluate HBE as a reaction descriptor under alkaline conditions.^52^ Recognizing that, apart from PGMs, most metals exhibit insufficient activity to enable direct comparison of hydrogen oxidation kinetics in alkaline electrolytes, they employed the HER as a probe reaction. By systematically measuring the exchange current densities of single-metal catalyst surfaces in alkaline media and correlating them with DFT-calculated HBE, they demonstrated that the HER exchange current density exhibits a characteristic volcano-type relationship with HBE. In this framework, Pt and Pd are located near the apex of the volcano, whereas transition metals such as Fe, Co, and Ni resided in the strong hydrogen-binding regime, and Cu, Ag, and Au fell on the weak-binding side. The activity differences spanned several orders of magnitude, and the overall trend closely resembled that observed in acidic electrolytes. Based on these results, the authors concluded that HBE can serve as an important reaction descriptor governing activity trend for hydrogen electrocatalysis (HOR/HER) in alkaline environments (Figure 3A).^52^ Building upon this foundation, Yan’s team further established a more direct correlation between HBE and catalytic reactivity through electrochemical measurements in 2015.^11^ Using cyclic voltammetry, they extracted experimental proxies for HBE on Pt surfaces across a wide pH range and systematically linked these values with pH-dependent HOR/HER activity, thereby constructing a verifiable causal relationship. By employing the same metal surface as a controlled platform, they showed that deviations of HBE from an optimal intermediate range lead to a pronounced deterioration in intrinsic HOR/HER kinetics. These findings indicate that HBE constitutes a primary energetic descriptor for hydrogen electrocatalysis on single-metal systems.

**Figure 3:**
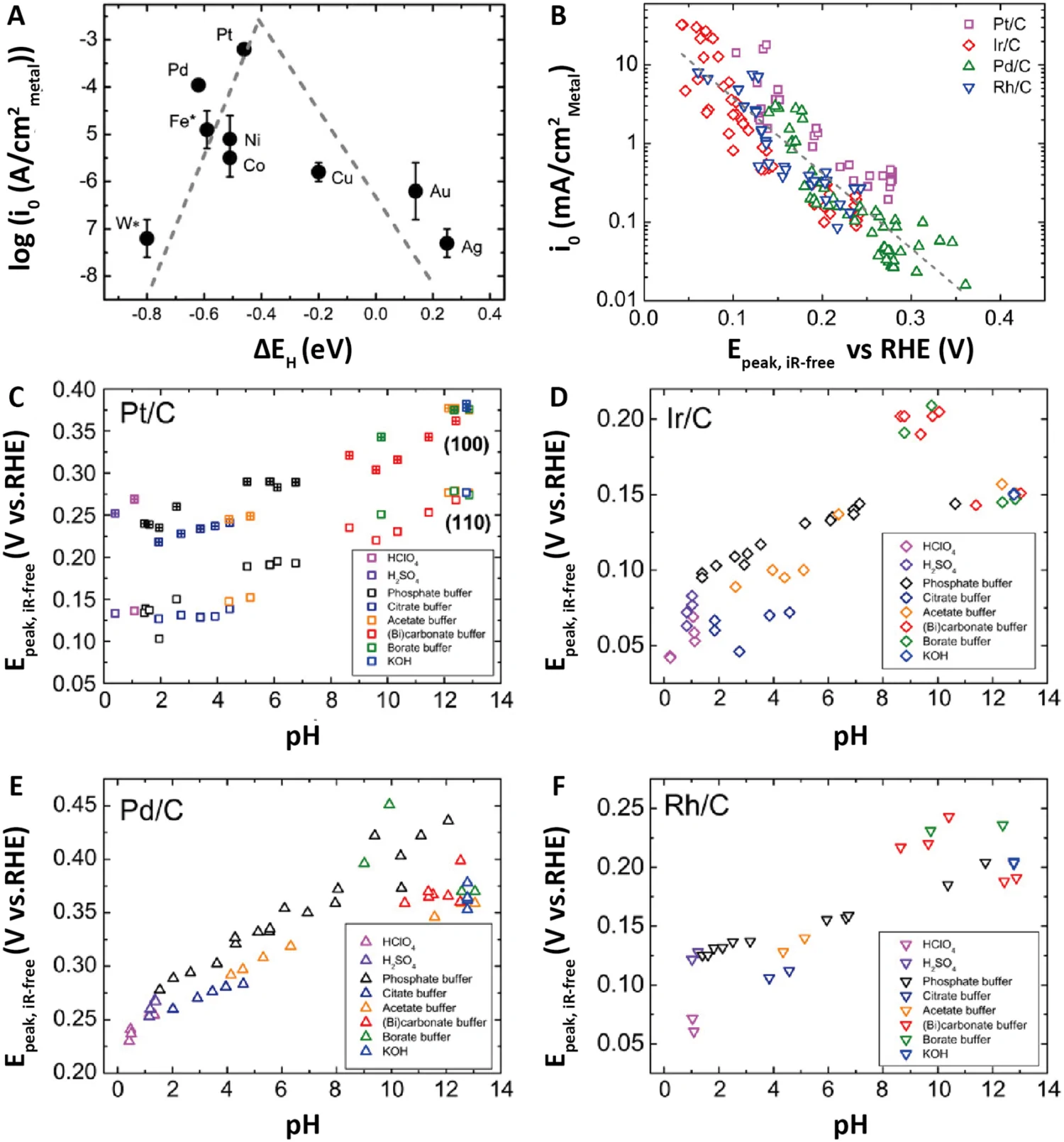
Correlations among hydrogen binding energy, H_upd_ features, and pH-dependent HOR/HER activity on monometallic catalysts. (A) Volcano-type relationship between the exchange current densities of monometallic surfaces and the calculated hydrogen binding energy.^52^ Reproduced with permission from ref 52. Copyright 2013 Royal Society of Chemistry. (B) Correlation between the exchange current densities of HOR/HER and the H_upd_ desorption peak potential for Pt/C, Ir/C, Pd/C, and Rh/C. (C–F) Variation of the H_upd_ desorption peak potentials with pH for (C) Pt/C, (D) Ir/C, (E) Pd/C, and (F) Rh/C.^53^ Reproduced from Zheng et al., Science Advances, DOI: 10.1126/sciadv.1501602 (2016), AAAS.

Related studies by Zheng et al. examined multiple PGM catalysts (Pt/C, Ir/C, Pd/C, and Rh/C) and identified a universal linear relationship between HBE and pH that is independent of metal identity.^53^ Desorption peaks associated with HUPD are commonly used as electrochemical indicators of HBE on catalytic surfaces. A positive shift in the hydrogen desorption peak generally reflects stronger HBE. As shown in Figure 3C–F, the hydrogen desorption potentials of PGM catalysts shift positively with increasing electrolyte pH, indicating strengthened hydrogen binding under more alkaline conditions. In parallel, Figure 3B shows that the exchange current densities (*j*_0_) are closely correlated with the hydrogen desorption peak potentials, confirming that changes in hydrogen adsorption strength are directly linked to HOR/HER kinetics in this PGM series. This interpretation is also consistent with the general volcano-type dependence of hydrogen electrocatalytic activity on HBE, in which either excessively strong or overly weak hydrogen binding leads to reduced activity. These observations support the use of HBE as a first-order activity descriptor for alkaline HOR on PGM surfaces, particularly when the compared catalysts share comparable reaction environments and similar elementary reaction steps. To further assess the role of hydroxyl species, CO stripping measurements are employed as an indirect probe of OH adsorption. The onset potential for CO oxidation shifted to lower values with increasing pH, indicating increasingly favorable hydroxyl adsorption. However, the intrinsic HOR/HER kinetics simultaneously deteriorated over the same pH range. This contrasting behavior suggests that enhanced OH adsorption does not translate into improved catalytic activity, thereby excluding hydroxyl species (OH_ad_) as the primary activity descriptor and reinforcing the central role of HBE in alkaline hydrogen electrocatalysis.

Beyond these systematic studies, investigations on distinct material systems further corroborate hydrogen binding energy as a key descriptor influencing alkaline HOR activity. In non-noble metal systems, Sheng et al. reported that multimetallic Ni-based catalysts, such as NiMo and CoNiMo, exhibit substantially enhanced HOR activity compared to pure Ni in alkaline electrolytes.^54^ Consistent trends observed from DFT calculations and H_2_ temperature-programmed desorption (H_2_-TPD) experiments indicate that multimetal alloying effectively weakens hydrogen binding on the catalyst surface. The close correspondence between reduced HBE and enhanced HOR activity demonstrates that modulating hydrogen adsorption is the primary driver for improved kinetics in these systems.

Overall, the studies above collectively establish HBE as a widely applicable first-order descriptor for rationalizing alkaline HOR activity across both single-metal and alloy catalysts. HBE is particularly useful when the compared catalysts share similar surface chemistry, interfacial environments, and elementary reaction steps, because it captures the balance among H_2_ activation, H* stabilization, and H_ad_ removal.^11,52,53^ In many systems, activity maxima consistently emerge when H adsorption is tuned toward a moderate regime, whereas deviations toward either strong- or weak-binding limits lead to pronounced kinetic losses, in line with Sabatier-type arguments. Nevertheless, the predictive capability of HBE is not universal. Deviations from HBE-based trends can arise when oxophilic components, potential-dependent oxygenated species, or interfacial water/electric double layer effects alter the effective reaction pathway.^24,45^ This limitation is particularly important in alkaline media, where the oxidative removal of H_ad_ can involves OH^–^, water, or surface oxygenated species, and where oxophilic motifs can make OH-related intermediates kinetically relevant under specific compositions, surface structures, and interfacial environments.^34,48,55^ Under such conditions, OHBE becomes an important complementary descriptor because it reflects the ability of oxophilic sites to stabilize OH_ad_ and facilitate OH_ad_-assisted H_ad_ removal. Therefore, OHBE does not replace HBE as a universal descriptor, but complements it when bifunctional site cooperation or OH_ad_-mediated pathways contribute significantly to the overall reaction rate.

### 2.3 Hydroxyl Binding Energy (OHBE) Theory (Bifunctional Theory)

In alkaline HOR, employing HBE as a sole descriptor is often insufficient to fully account for the kinetic limitations observed in basic media. Unlike acidic environments, alkaline HOR involves the active participation of hydroxyl ions (OH^–^) or surface-adsorbed OH_ad_.^24,48^ Consequently, the reaction kinetics are governed not only by the adsorption and dissociation of hydrogen intermediates but also by the affinity of the catalyst surface for oxygen-containing species.^25^ The OHBE has been increasingly recognized as a complementary descriptor to HBE, frequently discussed within a bifunctional mechanistic framework.^24,55^ Existing studies indicate that alkaline HOR rates are closely correlated with OH^–^ concentration and surface OH_ad_ coverage, suggesting that oxygen-containing intermediates are not merely spectator adsorbates but actively participate in the elementary reaction steps. In particular, the presence of OH_ad_ has been proposed to promote the Volmer step by facilitating the oxidation of adsorbed hydrogen intermediate from adjacent sites.^55^ Moreover, metal surfaces exhibiting higher oxygen affinity often display enhanced intrinsic activity in alkaline environments.^24,25,56,57^ Collectively, these observations indicate that OHBE becomes particularly relevant for catalysts containing oxophilic motifs or spatially coupled H/OH-binding sites, where alkaline HOR kinetics are constrained by the cooperative effects between hydrogen adsorption energetics and the formation of oxygen-containing intermediates at the catalyst surface.

Building on this understanding, Strmcnik et al. systematically investigated the role of surface oxophilicity and demonstrated that, even with comparable HBE, reaction rates vary markedly with the surface affinity toward OH_ad_.^24^ Their results revealed that the ability of catalyst to generate and stabilize OH_ad_ directly dictates the removal rate of hydrogen intermediates, thereby influencing the overall turnover frequency. These findings highlight that sufficient surface oxophilicity represents a requisite condition for facilitating the reaction in alkaline environments. To further quantify this impact, they compared various surfaces and found that Ir, despite possessing an HBE similar to Pt, exhibits a much higher oxygen affinity. The presence of OH_ad_ on Ir at low potentials results in an intrinsic HOR activity approximately 25-fold higher than that of Pt(111). As shown in Figure 4A–C, this effect is reflected in both single crystal and high surface area measurements, where Ir exhibits substantially higher HOR activity than Pt, and Pt(111) modified with Ni­(OH)_2_ adislands also shows enhanced activity relative to bare Pt(111) (Figure 4D). This demonstrates that tuning surface oxophilicity provides an effective route to overcoming kinetic bottlenecks in alkaline environments. Following this concept, the decoration of Pt(111) with highly oxophilic Ni­(OH)_2_ nanoclusters is shown to further promote the reaction kinetics through synergistic interactions at the interface between hydrogen-adsorbing and hydroxyl-adsorbing sites. Subsequent studies have reinforced this perspective.

**Figure 4:**
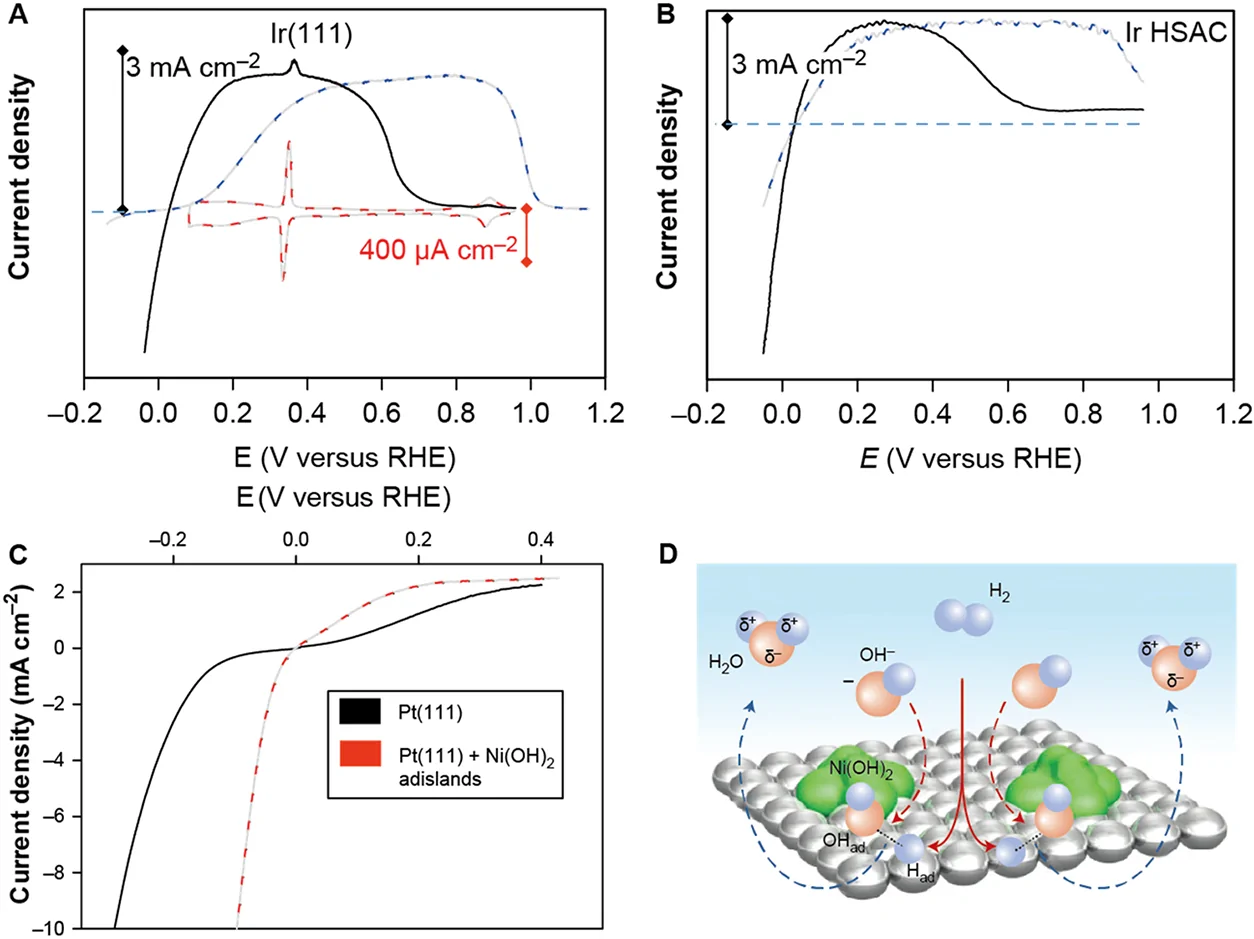
Representative electrochemical evidence and schematic illustration of the bifunctional mechanism in alkaline HOR. (A) HOR polarization curves and cyclic voltammograms of Ir(111) measured in 0.1 M KOH, with Pt(111) shown for comparison. (B) Comparison of HOR polarization curves of Pt (dash gray and blue) and Ir (black) high surface area catalysts. (C) HOR/HER polarization curves of Pt(111) and Pt(111) modified with Ni­(OH)_2_ adislands. (D) Schematic illustration of the proposed bifunctional effect on Ni­(OH)_2_/Pt­(111), in which Ni­(OH)_2_ provides sites for OH_ad_ adsorption and Pt provides sites for H_2_ dissociative adsorption and H_ad_ formation.^24^ Adapted with permission from Dusan Strmcnik et al. “Improving the Hydrogen Oxidation Reaction Rate by Promotion of Hydroxyl Adsorption.” Nature Chemistry 2013, 5, 300-306. Copyright 2013 Springer Nature.

More recent evidence also supports an OHBE-centered and bifunctional perspective. Li et al. employed *in situ* X-ray absorption spectroscopy (XAS) to reveal that activity enhancements in PtRu/C systems are decoupled from HBE, but are instead strongly associated with the presence of OH_ad_ on Ru sites within the HOR potential window (Figure 5A,B).^34^ The *in situ* Ru K-edge spectra and differential XANES analysis provide direct evidence for potential-dependent Ru–OH species under reaction-relevant conditions, indicating that Ru acts as an oxophilic site capable of stabilizing OH_ad_. Importantly, in the PtRu/C catalyst, Pt and Ru are integrated within the same catalytic ensemble, so Ru–OH motifs can be positioned in close proximity to Pt-based H-binding sites. This spatial arrangement provides spectroscopic support for atomic-scale site cooperativity, where Ru-associated OH_ad_ facilitates the oxidative removal of H_ad_ from neighboring Pt sites during the Volmer step. Therefore, the bifunctional enhancement should not be interpreted simply as a change in average adsorption energy, but as a local cooperative interaction between adjacent H-binding and OH-binding motifs. Similar behavior was also observed for Ru/C, further indicating that Ru-associated OH_ad_ plays a key role in accelerating alkaline HOR kinetics when suitable neighboring sites for hydrogen activation are available. Furthermore, Kim et al. established a volcano-type correlation between activity and OH-binding energy, demonstrating that excessively weak OHBE is insufficient to facilitate the generation of reactive OH-containing intermediates, whereas overly strong OHBE leads to surface passivation and site blocking (Figure 5C,D).^58^ This result further supports the importance of balanced OH adsorption in alkaline hydrogen electrocatalysis and has motivated subsequent efforts toward atomic-scale regulation of local coordination environments. For instance, Han et al. identified Ru–Ni diatomic sites as highly active centers where Ru facilitates hydroxyl adsorption while Ni promotes H_2_ dissociation (Figure 5E).^56^ Similarly, Wang et al. reported that embedding oxophilic rare-earth single atoms (e.g., La or Ce) into Pt nanoclusters increases the density of bifunctional sites for selective OH adsorption, thereby significantly accelerating the oxidative removal of adsorbed hydrogen (Figure 5F).^57^

**Figure 5:**
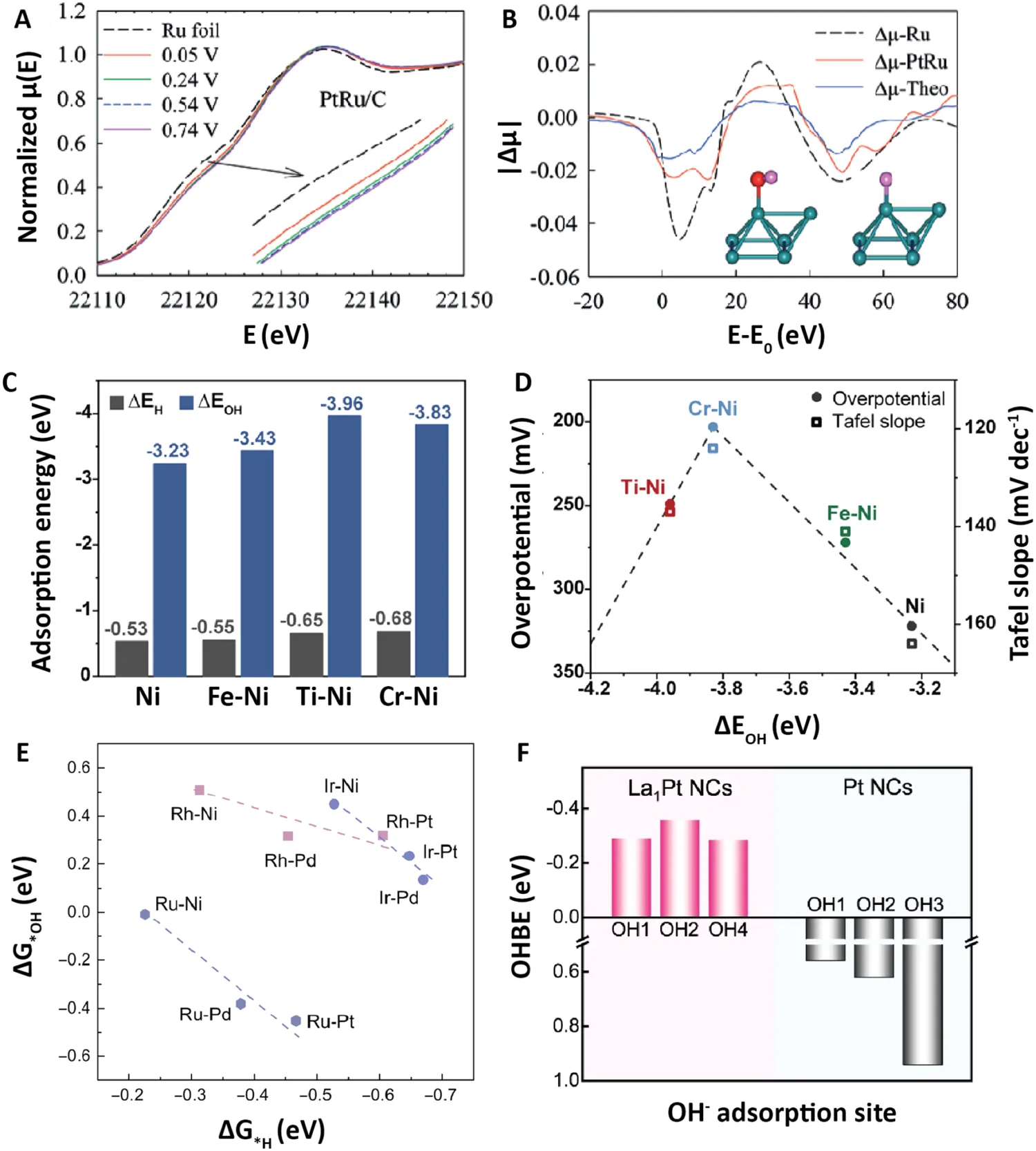
Spectroscopic and theoretical analysis of OH adsorption and its correlation with alkaline HOR activity. (A) Ru K-edge XANES spectra of Pt_1_Ru_1_/C collected in H_2_-saturated 0.1 M KOH at different applied potentials. (B) Experimental differential Δμ spectra derived from the XANES data, together with the calculated Δμ signal based on the model structures shown in the inset.^34^ Adapted with permission from ref 34. Copyright 2017 Wiley-VCH Verlag GmbH & Co. KGaA, Weinheim. (C) Calculated H-binding energies and OH-binding energies for Ni(111) and M-Ni(111) surfaces. (D) Volcano-type relationship of the overpotential at 10 mA cm^–2^ and Tafel slope as a function of Δ*E*_OH_ for M-Ni and Ni catalysts.^58^ Adapted with permission from ref 58. Copyright 2021 American Chemical Society. (E) Calculated ΔG_*H_ and ΔG_*OH_ values for different diatomic site catalysts (DASCs).^56^ Adapted from ref 56 under a Creative Commons CC BY License. (F) Comparison of the calculated OH binding energies at different adsorption sites on La_1_Pt NCs and Pt NCs.^57^ Adapted from ref 57 under a Creative Commons Attribution 4.0 International License.

Overall, these results highlight that alkaline HOR catalyst design cannot be guided by HBE or OHBE alone. Instead, high activity is achieved when HBE is tuned to an optimal, moderate regime to enable efficient H_2_ activation, while OHBE is simultaneously balanced to provide sufficient OH_ad_ for rapid oxidative removal of H_ad_ without inducing surface passivation or site blocking. Accordingly, practical design strategies increasingly focus on engineering heterogeneous, bifunctional motifs that spatially and energetically couple H-adsorbing and OH-adsorbing sites, including alloying, interfacial decoration, diatomic motifs, and single-atom additives. This dual-descriptor view provides a unified basis for rational materials selection and atomic-scale site design toward accelerated alkaline HOR kinetics. Nevertheless, HBE and OHBE primarily describe adsorbate–surface interactions and do not fully account for the role of the interfacial reaction environment.^45^ In alkaline media, the organization of interfacial water, hydroxide transport, cation-mediated interactions, and local EDL structure can further regulate the accessibility and stabilization of reaction intermediates.^22,47,48^ Therefore, a more complete understanding of alkaline HOR requires extending the descriptor framework from surface adsorption energetics to the interfacial environment, as discussed in the following section.

### 2.4 Effects of the Interfacial Environment

While HBE and OHBE offer useful descriptor-based frameworks for alkaline HOR, adsorption energetics alone do not fully capture the breadth of kinetic behavior observed in alkaline media.^45^ Even for catalysts with comparable binding strengths, variations in electrolyte cations and interfacial water organization can induce substantial rate differences, highlighting the critical role of the interfacial environment at the electrode–electrolyte boundary.^47,55^ These observations have motivated increasing attention to interfacial environment factors beyond intrinsic adsorption descriptors. Accordingly, this section examines the interfacial electric field, cation-specific effects, and interfacial water structure, and discusses how these factors couple with adsorption descriptors to ultimately govern alkaline HOR kinetics.^23^

#### 2.4.1 Effects of the Potential of Zero Free Charge

The *E*_pzfc_ is defined as the potential at which the excess electronic charge on the electrode surface vanishes, corresponding to a charge-neutral condition within the electrochemical double layer (EDL). Because surface free charge sets the interfacial electric field, *E*_pzfc_ provides a practical link between potential-dependent electrostatics, the orientation and hydrogen-bond network of interfacial water, and water-involved charge-transfer steps.^59,60^ Ledezma-Yanez et al. proposed that when the operating potential approaches *E*_pzfc_, the weakened interfacial electric field reduces the orientational bias of interfacial water, thereby lowering the solvent-reorganization penalty associated with interfacial charge transfer.^22^ This relationship is schematically illustrated in Figure 6, where increasing pH enlarges the separation between 0 V vs RHE and the pzfc, strengthens the interfacial electric field, and drives interfacial water toward a more ordered H-down configuration with a higher reorganization energy.^45,46^ In this view, the Volmer step is accelerated under weak-field conditions because a more labile interfacial water network can reorganize with a smaller energetic cost.

**Figure 6:**
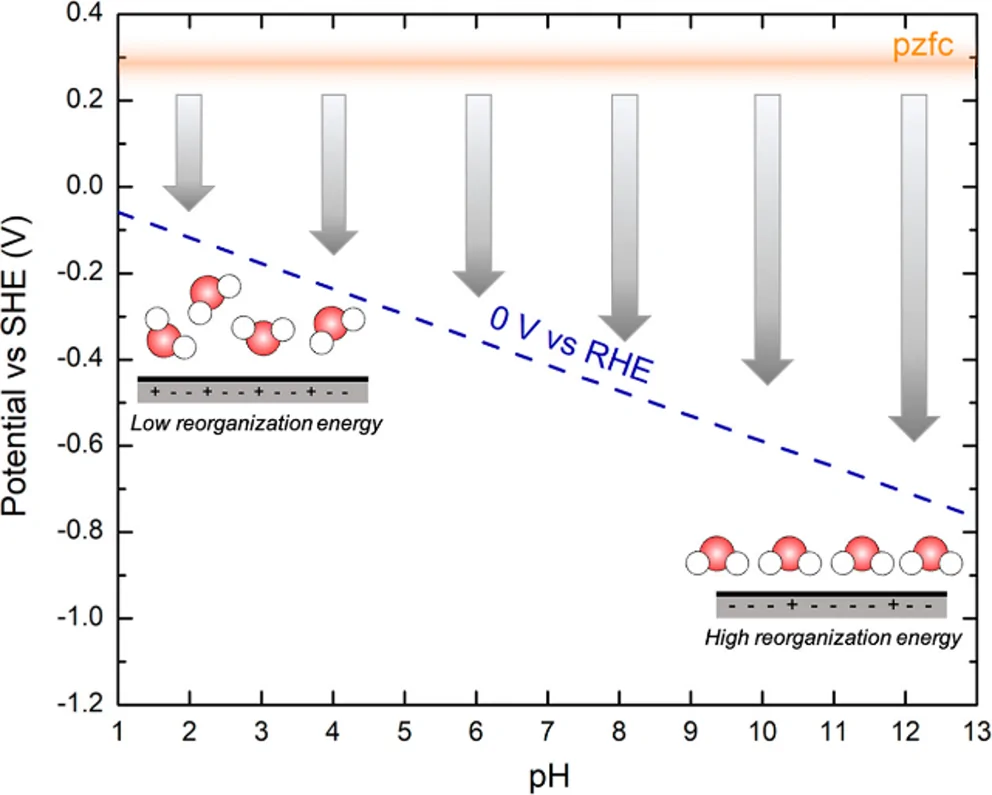
pH-dependent interfacial electric field, water orientation, and solvent reorganization at the electrode/electrolyte interface. Pourbaix diagram demonstrating the pH dependence of the hydrogen equilibrium potential (0 V vs RHE). Since the pzfc of Pt(111) is approximately constant on the SHE scale, the separation between the pzfc and 0 V vs RHE increases as pH increases, leading to a progressively more negative interfacial electric field under hydrogen-relevant potentials. As a result, interfacial water becomes increasingly oriented in an H-down configuration at high pH, accompanied by less facile water reorganization and a higher reorganization energy at the electrode–electrolyte interface. This trend illustrates why solvent organization and double-layer structure are expected to play a more important role in alkaline HER/HOR kinetics.^45^ Adapted with permission from ref 45. Copyright 2020 American Chemical Society.

Because *E*_pzfc_ is not directly measurable in most systems, it is commonly inferred from experimental proxies. CO charge-displacement can determine the potential of zero total charge (*E*_pzfc_); with *q*(*E*) analysis and model assumptions regarding specific adsorption and interfacial charge partitioning, *E*_pzfc_ can be estimated from the double-layer region.^59–63^ Alternatively, laser-induced temperature jump (LITJ) defines the potential of maximum entropy (PME), which often correlates with *E*_pzfc_ and is therefore used as a practical proxy.^61,63^ Furthermore, Fourier transform infrared-reflection absorption spectroscopy (FTIR-RAS) offers a molecular-level probe of interfacial electric field effects through potential-dependent vibrational Stark shifts and dipole reorientation of adsorbates and interfacial water, helping bracket the potential region associated with minimal net field.^63^ Within this framework, alkaline HOR kinetics can be sensitive to how far the operating potential lies from *E*_pzfc_. When potentials are referenced to the reversible hydrogen electrode (RHE, V_RHE_), *E*_pzfc_ is often reported to shift to more positive values with increasing pH, which can place the alkaline HOR operating window farther from the weak-field condition. A stronger interfacial field can enforce a more rigid water orientation and increase the effective solvent-reorganization barrier, raising the apparent barrier for the Volmer step and thereby suppressing intrinsic alkaline HOR rates.^62^

Taken together, the *E*_pzfc_ effect underscores that interfacial electrostatics and solvent structure can modulate activation barriers even when HBE and OHBE are comparable. For catalyst design, tuning *E*_pzfc_ toward the HOR operating window, or engineering local interfaces that effectively screen the interfacial field while preserving H- and OH-related adsorption motifs, offers a complementary route to mitigate solvent-reorganization constraints and improve alkaline HOR activity.

#### 2.4.2 Effects of Electrolyte Cations

Beyond the intrinsic properties of the electrode surface, alkaline HOR kinetics are strongly influenced by the identity and concentration of electrolyte cations. Alkali-metal cations (AM^+^) are now widely recognized as key modulators that reshape the EDL structure and the hydrogen-bond (H-bond) network of interfacial water.^55,64,65^ In particular, cation-induced changes in the interfacial water environment can substantially modulate the activation barrier for hydrogen or proton transport (PT) across the water network, thereby modulating the observed kinetics (Figure 7A–C).^66^

**Figure 7:**
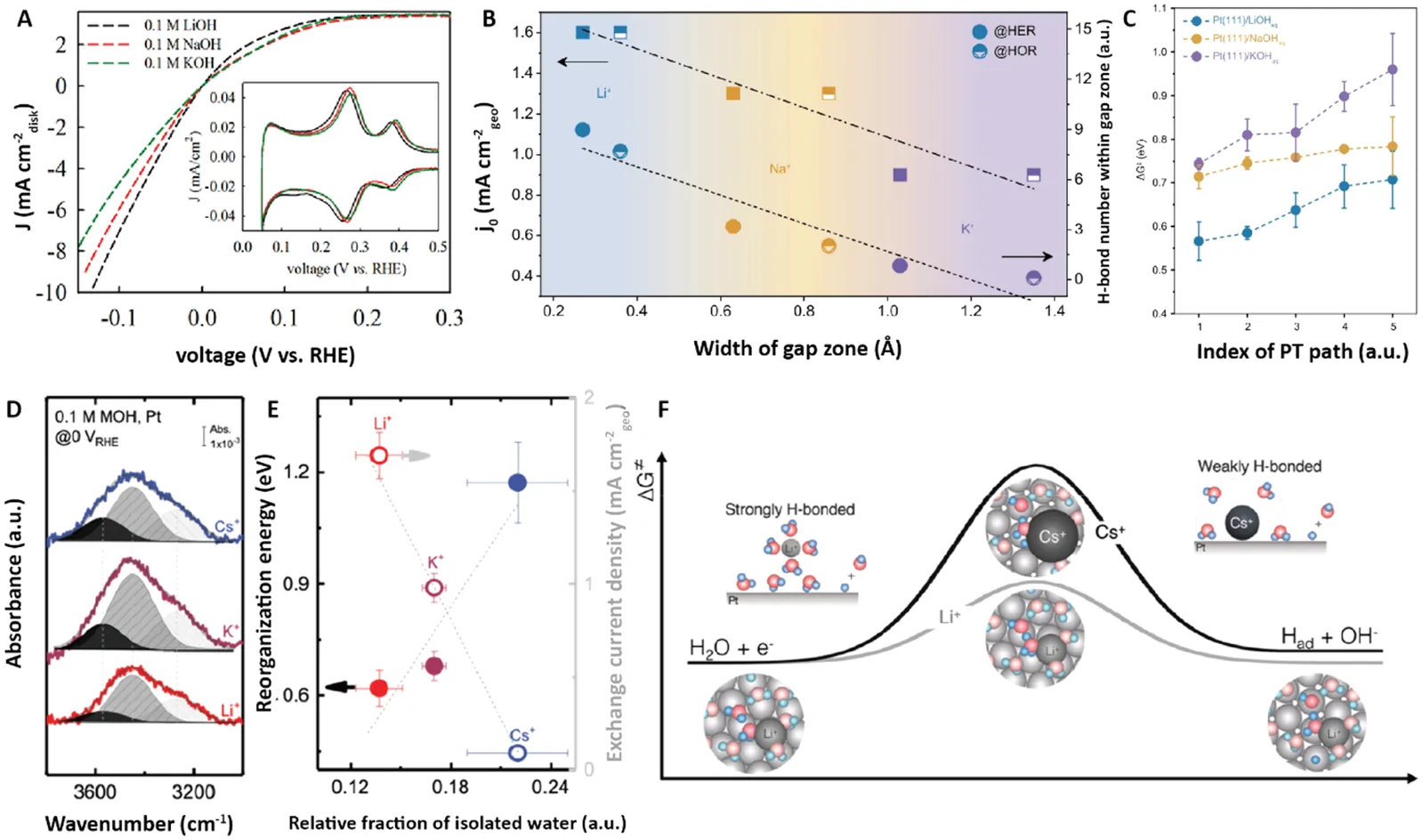
Cation-dependent interfacial water structure and proton-transfer characteristics in alkaline hydrogen electrocatalysis. (A) HER/HOR polarization curves of polycrystalline Pt in H_2_-saturated 0.1 M LiOH, NaOH, and KOH, with the corresponding CV curves shown in the inset.^48^ Adapted with permission from ref 48. Copyright 2019 American Chemical Society. (B) Experimental exchange current densities and the theoretical H-bond number within the interfacial gap zone plotted as a function of gap-zone width. (C) Free-energy barriers for proton transfer across the interfacial electric double layer in different Pt(111)/AMOH_aq_ systems.^66^ Adapted from ref 66 under a Creative Commons Attribution-NonCommercial-NoDerivatives 4.0 International License. (D) Deconvoluted OH stretching bands measured at 0 V vs RHE, showing water populations with three kinds of hydrogen-bonding configurations. (E) Reorganization energies and exchange current densities plotted against the relative fraction of isolated interfacial water in various alkaline electrolytes. (F) Schematic illustration of the proposed cation-dependent HER/HOR mechanism, highlighting the influence of cation solvation on interfacial water structure and proton-transfer kinetics.^47^ Adapted from ref 47 under CC-BY-NC-ND 4.0.

Current mechanistic interpretations of cation effects generally converge on two complementary perspectives. The first emphasizes noncovalent interactions between hydrated cations and chemisorbed OH_ad_ at the interface.^35,64,65^ Danilovic et al. suggested that high charge-density hydrated cations can couple to surface OH_ad_ through their hydration shells, forming interfacial OH_ad_···AM^+^ (H_2_O)_*x*_ adducts.^67^ Such interactions can promote water dissociation, alter the adsorption and removal of OH-related species in alkaline HER. In alkaline HOR, however, analogous cation-associated interfacial structures may, depending on coverage and potential, contribute to site blocking and reduce the number of accessible sites for H_2_ adsorption and dissociation, thereby inhibiting HOR kinetics.^48,67,68^

The second perspective focuses on kinetic cation effects, attributing rate variations primarily to cation-dependent constraints on proton or hydrogen transport within the EDL.^69^ By combining ab initio molecular dynamics (AIMD) simulations with *in situ* attenuated total reflection surface-enhanced infrared absorption spectroscopy (ATR-SEIRAS), Li et al. showed that size-dependent cation accumulation at the interface reorganizes the hydration structure.^66^ Excess accumulation of larger hydrated cations disrupts the continuity of the H-bond network and increases the free-energy barrier for transport from the bulk electrolyte to the reactive interface.

Huang et al. systematically measured the exchange current density (*j*_0_) for HOR/HER on Pt across a range of pH values in electrolytes containing different alkali-metal cations (Li^+^, Na^+^, K^+^, Rb^+^, Cs^+^).^47^ The alkaline kinetics are strongly cation-dependent, following the trend Li^+^ > Na^+^ > K^+^ > Rb^+^ > Cs^+^. Within the Marcus–Hush-Chidsey (MHC) framework, the fitted interfacial reorganization energy (λ) increased systematically with cation size, while the fitted entropy-related term decreased, consistent with cation-dependent differences in interfacial solvation and accumulation. In this interpretation, cations often classified as structure-breaking in aqueous media (e.g., Cs^+^) tend to accumulate at the interface and undergo partial desolvation, displacing interfacial water and weakening the H-bond network. In contrast, structure-making cations (e.g., Li^+^) better retain their hydration shells, allowing the interface to maintain a more continuous water layer and H-bond structure. Consistent with this picture, *in situ* SEIRAS analysis deconvoluted the OH-stretching band at 0 V vs RHE into three components (∼3570, ∼3450, and ∼3270 cm^–1^), assigned to isolated-water, asymmetrically H-bonded water, and symmetrically H-bonded water, respectively, enabling a quantitative descriptor of interfacial water structure. The spectroscopic basis for this analysis is shown in Figure 7D,E. Notably, *j*_0_ decreased approximately linearly with increasing isolated-water fraction. Together, these results link cation-dependent interfacial water structure to λ and *j*_0_, providing a molecular-level basis for how alkali-metal cations modulate alkaline HOR kinetics by reshaping the interfacial solvent environment (Figure 7F).

In essence, electrolyte cations can exert both promoting and inhibiting influences in alkaline HOR. They not only modulate the effective coverage of interfacial species and the barriers associated with interfacial water reorganization via noncovalent interactions, but also regulate the interfacial electric-field environment and transport pathways within the EDL, collectively shaping alkaline HOR kinetics.

#### 2.4.3 Effects of Interfacial Water Structure

Under alkaline conditions, water molecules are no longer merely an inert reaction medium; rather, the structure and dynamics of interfacial water have emerged as key determinants of electrocatalytic kinetics. In this context, alkaline HOR kinetics are often not fully captured by a single descriptor. Instead, they correlate strongly with interfacial-water distribution and orientation,^70–73^ hydrogen-bond network connectivity,^38,45,74^ and electric-field–dependent charge-transfer characteristics within the EDL.^49,63,75^

Liu et al. further proposed that the rate-determining step (RDS) of alkaline HOR is governed by interfacial water, rather than solely by the strength of the Pt–H bond.^23^ Using kinetic isotope effect (KIE) measurements on the Pt polycrystalline electrode, they compared HOR and HER polarization behavior in H_2_-saturated 0.1 M HClO_4_ and 0.1 M KOH prepared with either H_2_O or D_2_O. In acidic 0.1 M HClO_4_, the HOR curves measured from −0.02 to 0.06 V vs RHE showed only a minor difference between H_2_O and D_2_O, consistent with the reaction being primarily limited by H_2_ diffusion under this condition. In contrast, in alkaline 0.1 M KOH, the HOR/HER curves recorded from −0.2 to 0.3 V vs RHE showed markedly slower kinetics in D_2_O than in H_2_O. This showed that isotope-sensitive interfacial water or hydroxide-associated processes become important in alkaline media. This contrast supports the view that interfacial water actively participates in shuffling reaction intermediates across the electrode–electrolyte interface during alkaline HOR. They further employed *in situ* XAS to identify interfacial water-related species under operating potentials, providing direct experimental evidence that the structure of interfacial water can modulate the alkaline HOR pathway. In Ru-modified Pt, Fourier transformed extended X-ray absorption fine structure (FT-EXAFS) revealed a Ru–O coordination feature with a bond length of ∼ 2.03 Å at low potentials. This feature is consistent with Ru–H_2_O and is attributed to bonding between metallic Ru^0^ and the oxygen atom of specifically adsorbed, oxygen-down interfacial water (H_2_O↓_ad_) (Figure 8A). Notably, this Ru^0^-H_2_O↓_ad_ signature appears only at low potentials and vanishes as Ru is progressively oxidized to Ru­(OH)_*x*_; concurrently, the HOR-promoting effect of Ru on Pt diminishes. This potential-dependent correspondence indicates that HOR promotion is closely associated with the orientation and bonding state of specific interfacial water species, which can participate in regulating intermediate transfer and reaction pathways, thereby making interfacial water structure a key factor governing the intrinsic kinetics of alkaline HOR (Figure 8B).

**Figure 8:**
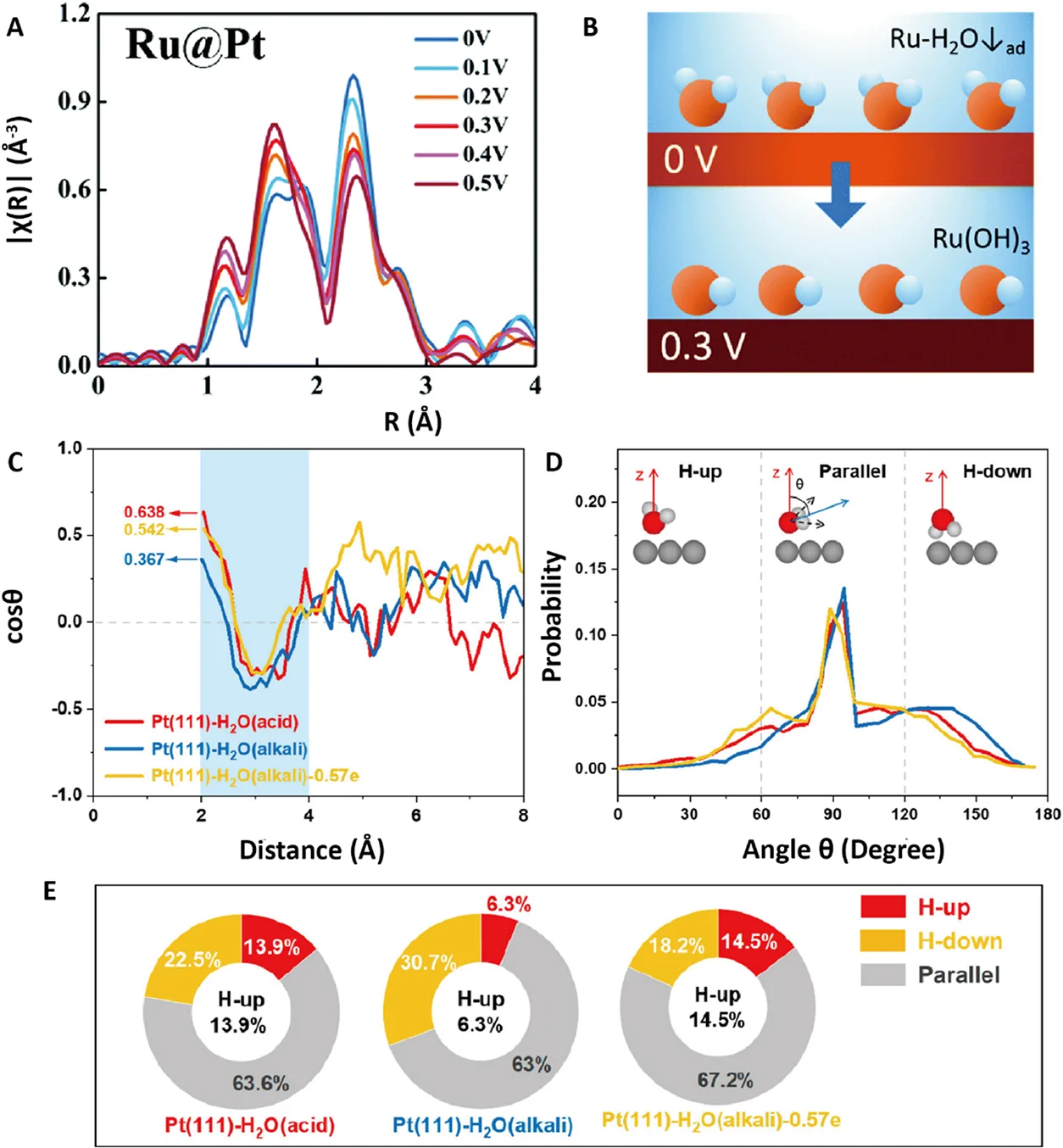
Interfacial water adsorption configurations and their variations across different electrochemical environments. (A) *In-situ* FT-EXAFS spectra of Ru@Pt collected at different applied potentials. (B) Schematic illustration of the redox transformation from Ru–H_2_O↓_ad_ to Ru­(OH)_3_ with increasing potential.^23^ Reproduced with permission from ref 23. Copyright 2020 Royal Society of Chemistry. (C) Variation of *cos θ* as a function of the Pt(111)-water distance for Pt(111)-H_2_O in acid, Pt(111)-H_2_O in alkali, and Pt(111)-H_2_O in alkali with 0.57e. (D) Angular distributions of interfacial water orientations within the Pt-water distance range of 2–4 Å. (E) Relative fractions of interfacial water molecules with different orientations.^76^ Adapted with permission from ref 76. Copyright 2022 American Chemical Society.

Complementarily, Li et al. used AIMD and DFT to quantify structural differences in the interfacial water layer on Pt(111) across acidic and alkaline environments.^76^ Based on the Pt–O pair correlation function, they distinguished an adsorbed water layer in the inner Helmholtz plane (IHP) from an interfacial water layer in the outer Helmholtz plane (OHP). The authors further reported that under alkaline conditions the OHP becomes narrower and the population of water in the IHP decreases, accompanied by a smaller work function (WF). In addition, their orientation analysis showed an increased fraction of the H-down configuration in alkaline media; accordingly, they suggested that such an interfacial arrangement can stabilize H_ad_ and impede its desorption and transfer, thereby hindering alkaline HOR (Figure 8C–E).

Beyond computational approaches, in situ vibrational spectroscopy has provided complementary molecular-level evidence for interfacial water regulation during alkaline HOR. The O–H stretching features of interfacial water are sensitive to water adsorption strength, hydrogen-bonding configuration, and local electric-field response, allowing *in situ* Raman and infrared (IR) spectroscopies, particularly surface-enhanced Raman spectroscopy (SERS) and SEIRAS, to track potential-dependent water reorganization under electrochemical conditions and correlate these structural changes with HOR kinetics.^72,73^ Wu et al. further demonstrated that *in situ* Raman spectroscopy can probe interfacial water adsorption during alkaline HOR.^77^ For d-p hybridized Ru catalysts, Sn–Ru/C showed stronger O–H stretching intensity than Ru/C at 0 V, indicating enhanced interaction with interfacial water molecules. Potential-dependent Raman spectra revealed broad O–H stretching bands around 3200 and 3400 cm^–1^, which showed a blue shift with increasing potential due to the Stark tuning effect (Figure 9A). The larger Stark slopes of different O–H stretching modes on Sn–Ru/C compared with Ru/C further indicated stronger interfacial water adsorption (Figure 9B). Combined with DFT results, these Raman observations suggest that Sn-induced d-p hybridization promotes interfacial water adsorption, thereby accelerating alkaline HOR kinetics.

**Figure 9:**
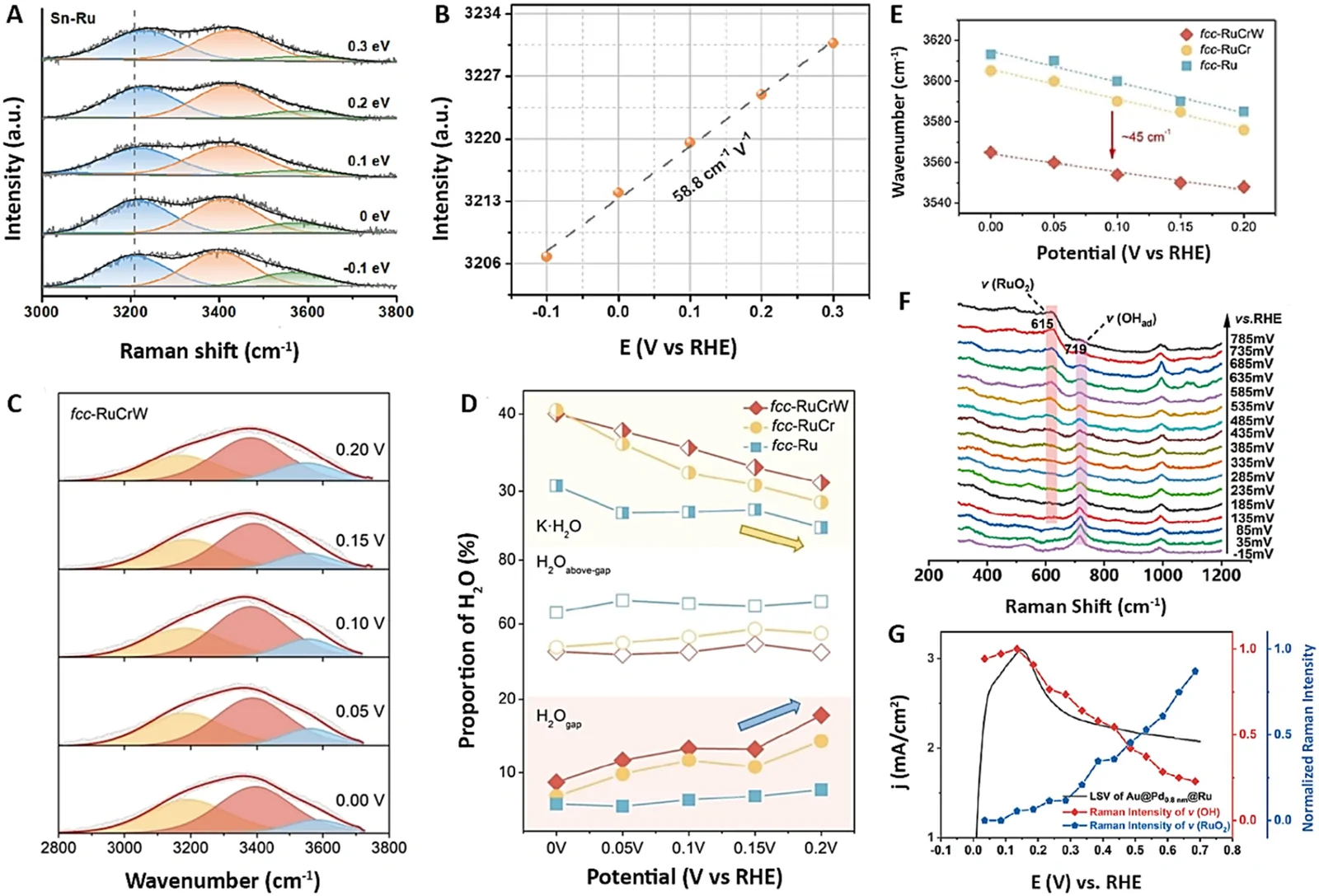
*In-situ* vibrational spectroscopic evidence for interfacial water regulation and oxygenated intermediates during alkaline HOR. (A) Potential-dependent Raman spectra of Sn–Ru/C, showing O–H stretching features of interfacial water. (B) Stark tuning behavior of the O–H stretching mode, reflecting potential-dependent interfacial water adsorption.^77^ Adapted with permission from ref 77. Copyright 2023 American Chemical Society. (C) *In-situ* SEIRAS spectra of fcc-RuCrW with deconvoluted O–H stretching bands from different water populations. (D) Potential-dependent distribution of K·H_2_O, H_2_O_above‑gap_, and H_2_O_gap_ in the EDL. (E) O–H stretching frequency shift of H_2_O_gap_ on fcc-Ru-based catalysts, indicating H-bond network modulation.^78^ Adapted with permission from ref 78. Copyright 2024 Wiley-VCH GmbH. (F) Potential-dependent *in situ* SERS spectra of RuO_2_ and OH_ad_ features on Au@Pd@Ru. (G) Correlation between HOR polarization behavior and Raman intensities of OH_ad_ and RuO_2_.^79^ Adapted with permission from ref 79. Copyright 2023 American Chemical Society.

Complementarily, Yue et al. employed *in situ* SEIRAS to resolve the potential-dependent interfacial water structure on fcc-Ru-based catalysts during alkaline HOR.^78^ By deconvoluting the O–H stretching region, they distinguished three water populations in the EDL, including H_2_O_gap_ (∼3600 cm^–1^), H_2_O_above‑gap_ (∼3400 cm^–1^), and K·H_2_O (∼3200 cm^–1^) (Figure 9C). The fcc-RuCrW catalyst showed a higher H_2_O_gap_ proportion than fcc-RuCr and fcc-Ru, indicating increased water concentration in the gap region and enhanced H-bond network connectivity. With increasing potential, the H_2_O_gap_ fraction increased while the K·H_2_O fraction decreased, suggesting potential-driven redistribution of interfacial water from the electrode-proximal region to the EDL gap region (Figure 9D). In addition, the ∼45 cm^–1^ red shift of the H_2_O_gap_ O–H stretching band on fcc-RuCrW relative to fcc-Ru indicates weakened O–H bonds and stronger hydrogen bonding among water molecules (Figure 9E). The observed correlation between OHBE and H_2_O_gap_ proportion further suggests that enhanced OH adsorption can reconstruct the interfacial water environment and facilitate hydrogen transfer across the EDL, thereby improving alkaline HOR kinetics.

In addition to probing water-related structures, vibrational techniques have also been used to identify oxygenated intermediates and to support bifunctional mechanisms.^37^ Song et al. employed *in situ* SERS on Au@Pd@Ru core–shell model catalysts and directly observed RuO_2_ and OH_ad_ peaks at 615 and 719 cm^–1^, respectively, with the OH_ad_ assignment verified by deuterium isotope experiments (Figure 9F). The OH_ad_ intensity decreased with increasing potential and closely correlated with the HOR oxidation current, indicating its role as a key reactive intermediate (Figure 9G).^79^

Taken together, these studies highlight that interfacial water is not a passive spectator in alkaline HOR but an active, potential-dependent component of the reaction environment that can modulate both intermediate stability and charge-transfer pathways. The key implication is that alkaline HOR kinetics depend not only on adsorption descriptors (HBE and OHBE) but also on how the interface organizes water, including its orientation, hydrogen-bond connectivity, and local electric-field response. From a materials-design perspective, catalysts and promoters that stabilize a favorable interfacial water motif within the HOR potential window, while avoiding interfacial dehydration or overly rigid water ordering, are more likely to sustain rapid H_ad_ turnover. Accordingly, interfacial water structure provides an additional, experimentally accessible handle for rationally tuning alkaline HOR activity beyond conventional adsorption-energy optimization.

At the same time, computational descriptions of alkaline HOR should be interpreted with caution. Many DFT and AIMD studies still rely on idealized slab models, simplified surface terminations, limited adsorbate coverages, finite simulation cells, and simplified solvent/electrolyte representations, which cannot fully capture realistic alkaline electrode–electrolyte environments.^80,81^ In particular, explicit AIMD can provide molecular-level insight into interfacial water and ions, but is often limited by accessible time scales, simulation-cell sizes, ion diffusion, and the difficulty of representing the full EDL. Conversely, implicit or hybrid solvation models improve computational efficiency and enable treatment of capacitive charging, but they usually rely on coarse-grained electrolyte descriptions and parametrized interfacial responses.^80,81^ As a result, alkali-metal cation distributions, H-bond networks, interfacial electric fields, local reaction environments, and solvent reorganization can substantially alter adsorption energies and elementary reaction barriers.^82,83^ Moreover, most calculations are based on static or idealized surface models, whereas multimetallic catalysts may reconstruct, segregate, oxidize, hydroxylate, or leach under operating potentials, making the true active interface different from the modeled structure. Therefore, DFT-derived HBE, OHBE, and reaction barriers should be regarded as mechanistic guidelines rather than absolute predictors, and should be validated by *in situ* spectroscopy and electrochemical measurements.^81,82^

### 2.5 Quantitative Metrics for Benchmarking Alkaline HOR

To compare alkaline HOR catalysts across studies, activity should be reported with clearly defined normalization, consistent kinetic extraction, and explicit checks for mass transport and ohmic losses. In practice, quantitative benchmarking commonly relies on geometric current density (*J*_geo_), ECSA-normalized specific activity (*J*_s_), mass activity (*J*_m_), kinetic current density (*J*_k_), exchange current density (*J*_0_), and Tafel slope. Reporting these metrics together, alongside key protocol details that govern transport, resistance, and electrode architecture, helps distinguish intrinsic catalytic kinetics from apparent performance differences introduced by catalyst utilization, H_2_ mass transport, and measurement conditions.

#### 2.5.1 Geometric, Specific, and Mass-Normalized Activity

Geometric current density (*J*_geo_), normalizes the measured current (*i*) by the geometric electrode area (*A*_geo_), with *J*_geo_ equal to *i* divided by *A*_geo_. This metric provides an intuitive, device-relevant readout and is convenient for cross-comparison because the geometric area is unambiguous. Many studies report the *J*_geo_ at a small overpotential, often at η equal to 50 or 100 mV, where the forward HOR branch approaches limiting current region. However, *J*_geo_ inherently convolutes intrinsic kinetics with mass transport and catalyst-layer effects, including wetting, porosity, ionomer distribution, catalyst-layer thickness, and local hydrogen supply. As a result, *J*_geo_ alone is not sufficient to claim intrinsic activity enhancements unless transport and electrode architecture are demonstrated to be comparable.

Specific activity, *J*_s_, normalizes current by the electrochemically active surface area (ECSA), with *J*_s_ equal to *i* divided by *A*_ECSA_.^6^ This normalization better reflects the activity per accessible surface site and is therefore widely used to compare catalysts with different particle sizes and dispersions. Nevertheless, *A*_ECSA_ extraction can be method dependent, particularly for alloys, oxophilic surfaces, and catalysts prone to surface segregation or reconstruction in alkaline media. It is thus essential to state explicitly how *A*_ECSA_ is obtained (e.g., HUPD, CO stripping, CuUPD, or oxide reduction) and to clarify the charge-to-area conversion assumptions and any baseline corrections (e.g., carbon double-layer contribution if capacitance-based approaches are used).

Mass activity, *J*_m_, normalizes current by catalyst mass (often per total catalyst mass or per precious metal mass). This metric is central for cost-constrained catalyst design and for assessing utilization in low-PGM formulations. However, mass activity is strongly influenced by dispersion and accessibility; accordingly, it is recommended to be reported together with ECSA-based metrics to distinguish genuine improvements in intrinsic surface kinetics from apparent gains driven mainly by higher utilization.

#### 2.5.2 Kinetic Current and Mass Transport Correction

In rotating disk electrode (RDE) measurements, the measured current density contains both kinetic and diffusion contributions and is often corrected using the Koutecky–Levich (K–L) equation:^6,25,84–86^

$$\frac{1}{J}=\frac{1}{J_{k}}+\frac{1}{J_{d}}=\frac{1}{J_{k}}+\frac{1}{B\omega^{1/2}}=\frac{1}{J_{k}}+\frac{1}{0.62\mathrm{nF}D^{2/3}V^{-1/6}C_{0}\omega^{1/2}}$$

where *J*_k_ is the kinetic current density, and *J*_d_ is the diffusion-limited current density. *B* represents the Levich constant, *C*_0_ is the solubility of H_2_, *F* is the Faraday constant, *D* is the H_2_ diffusion coefficient, *ν* is the kinematic viscosity, and ω is the rotating rate. For a given catalyst, the K–L equation reveals that *J*_d_^–1^ is linear to ω^–1/2^ with the slope of reciprocal of Levich constant (B). Reporting *J*_k_ generally enables fairer cross-study comparisons because it reduces the direct influence of rotation rate and diffusion constraints, thereby emphasizing intrinsic reaction kinetics.

The K–L equation is used to remove a defined transport-limited contribution from measured currents to estimate *J*_k_. This equation is derived from the reactions that exhibit a first-order dependence on the gas-phase reactant. However, alkaline H_2_ oxidation can deviate from simple first-order behavior with respect to H_2_ partial pressure, so the validity of a direct K–L analysis should be evaluated carefully. In practice, K–L correction is still often applied as an operational approach to remove a well-defined transport contribution, but it should be restricted to the regime where transport effects remain modest. To minimize error amplification, we recommend performing K–L correction only when the measured current remains below ∼80% of the HOR diffusion-limited current, *J*_d_ (a guideline generally applicable in alkaline media). As *J* approaches *J*_d_, small uncertainties in *J*_d_ and *J* propagate strongly, causing the error and uncertainty in the K–L-derived *J*_k_ to increase rapidly near the transport-limited plateau.

#### 2.5.3 Exchange Current Density and Low Overpotential Benchmarking

Once the kinetic current density is reliably extracted (with appropriate *iR* and mass-transport corrections), the exchange current density (*J*_0_) can be determined by fitting the near-equilibrium region to the Butler–Volmer (B–V) equation:^6,25,84–86^

$$J_{k}=J_{0}\left[e^{\left(\frac{\alpha F}{\mathrm{RT}}\right)\eta }-e^{\left(\frac{-\left(1-\alpha \right)F}{\mathrm{RT}}\right)\eta }\right]$$

where *J*_k_ is the kinetic current density, *J*_0_ is the exchange current density, α is the transfer coefficient, *F* is Faraday’s constant, *R* is the universal gas constant, *T* is the temperature in Kelvin, and η is the overpotential. *J*_0_ and α can be extracted by fitting this B–V equation, and an optimal fit for Pt/C should yield a symmetric curve with apparent α ≈ 0.5, consistent with previous reports.^6,25,84–86^

Butler–Volmer equation can be simplified in the micropolarization (MR) region, typically within an overpotential window of −10 to +10 mV. By applying a Taylor expansion, the exponential terms reduce to a linear form, and the Butler–Volmer relation can be expressed as

$$J_{k}=J_{0}\left(\frac{\eta F}{\mathrm{RT}}\right)$$

By plotting the kinetic current density against overpotential, the slope in the micropolarization region (MR) can be used to determine the exchange current density (*J*_0_). The *J*_0_ obtained from the Butler–Volmer fitting (B–V) and from the MR linear region method should be comparable, providing a reasonable consistency check.^6,86^

For practical benchmarking, because HOR is fast on many PGM-based catalysts, the extracted *J*_0_ can be sensitive to uncompensated resistance and residual transport limitations, even when the polarization curve appears well-behaved. For meaningful comparisons, *iR* correction and adequate hydrogen delivery are essential, and the analysis window should be chosen to minimize artifacts from transport losses. When this metric is used, it is important to specify whether the value is transport corrected and to state the normalization basis, either geometric or ECSA normalized, because these choices can change the interpretation of activity trends across different electrode configurations.

#### 2.5.4 Tafel Slope Analysis

Tafel analysis is widely used to summarize how the kinetic current varies with overpotential and to provide phenomenological insight into alkaline HOR mechanisms. In practice, the Tafel slope is obtained either from a plot of η versus log­(|*J*_*k*_|) or from Butler–Volmer (B–V) fitting through the transfer coefficient (α).^85^ To ensure correct physical meaning, the analysis should be performed in a potential window where mass-transport contributions are negligible or rigorously corrected, and ohmic losses are *iR*-compensated.^84^

The mechanistic interpretation for HOR is often discussed within two elementary-step sequences, Tafel–Volmer and Heyrovsky–Volmer, which together give four common rate-controlling cases (Table 1).^42,43,85^ When the Volmer step is rate-determining in a Tafel–Volmer sequence, HOR is expected to show the same absolute Tafel slope (∼118 mV dec^–1^ at 298 K) and a symmetric B–V response (frequently consistent with an apparent α ≈ 0.5 under symmetric assumptions). In contrast, for the Heyrovsky–Volmer pathway, the B–V behavior can become asymmetric, and the apparent Tafel slopes differ depending on whether Volmer or Heyrovsky limits the rate; a commonly cited pair is ∼118 and ∼39 mV dec^–1^ for HOR, reflecting whether the electron-transfer event occurs effectively before or after the rate-limiting step and how coverage terms enter the measured kinetics.

**Table 1:** Tafel Slope and Kinetic Expressions for HOR

HOR mechanism	Tafel (RDS) – Volmer	Tafel–Volmer (RDS)	Heyrovsky (RDS) – Volmer	Heyrovsky–Volmer (RDS)
Tafel slope at 25 °C	2.303RT/2F = ∼29 mV dec^–1^	2.303RT/[(1−α)F] = ∼118 mV dec^–1^	2.303RT/[(1−α)F] = ∼118 mV dec^–1^	2.303RT/[(2−α)F] = ∼39 mV dec^–1^

Importantly, Tafel slopes should not be used as a standalone proof of mechanism. Different pathways can yield similar slopes within experimental uncertainty, and apparent slopes are easily biased if the selected window is not in a true Tafel regime or is affected by residual transport. This is exemplified by HOR on Pt in acid, where conventional RDE measurements can be dominated by H_2_ mass-transfer limitation, producing an apparent Tafel-like trend that does not represent intrinsic charge-transfer kinetics. The same caution applies to alkaline HOR as currents approach the diffusion-limited plateau: small uncertainties in the limiting current, *iR* correction, or gas delivery (e.g., bubbles) can strongly distort both K–L correction and subsequent slope extraction. Therefore, robust benchmarking should interpret Tafel slopes together with complementary checks, such as multirotation-rate diagnostics, consistency between HER and HOR slopes on the same catalyst, and micropolarization/B–V fitting near equilibrium. Finally, because alkaline HOR couples H_2_ adsorption/dissociation, charge transfer, and OH^–^/H_2_O-mediated interfacial interactions, the Tafel slope can vary with potential, catalyst composition, surface coverage, and electrolyte, and should be reported with explicit fitting windows and correction procedures.

#### 2.5.5 Durability Test

Beyond initial activity metrics, alkaline HOR catalysts should be evaluated using durability protocols that capture activity retention, elemental leaching, and structural evolution under electrochemical stress. In practice, durability is commonly assessed by accelerated durability tests (ADTs) based on potential cycling within a defined window under H_2_ to mimic HOR-relevant conditions. Alternatively, chronoamperometry can be performed at a representative HOR potential (e.g., 0.1 V vs RHE) to monitor current decay over time, or chronopotentiometry at a fixed current can be used to probe steady-state degradation. In addition to these electrochemical stress tests, poisoning sensitivity should be systematically evaluated. A commonly used protocol in the HOR field is to introduce CO into the H_2_ feed (e.g., 1000 ppm of CO) and combine it with chronoamperometry to track the deactivation rate, thereby quantifying CO tolerance and operational robustness.

For meaningful comparisons, durability results should be reported together with clearly defined test parameters (electrolyte identity/concentration, gas environment, temperature, scan rate, potential limits, cycle number, catalyst loading) and quantified using consistent pre/post-test benchmarks, such as shifts in HOR polarization curves and the retention of key kinetic metrics (e.g., *J*_0_, ECSA-normalized specific activity, and mass activity). Importantly, stability analysis should not rely solely on current retention. To elucidate degradation pathways, elemental leaching should be verified by analyzing the post-test electrolyte (e.g., ICP-OES/ICP-MS) and comparing pre/post-composition at the catalyst (EDS/XPS), while structural evolution (particle coarsening, surface segregation, oxidation/passivation of oxophilic motifs, and support/ionomer-induced changes) should be tracked by post-mortem and, where possible, *operando* characterization (TEM, XRD, and XAS).

It should also be emphasized that most durability and poisoning-tolerance evaluations for alkaline HOR catalysts are currently performed in RDE half-cell systems. These measurements are valuable as initial diagnostic tests because they allow well-controlled comparison of intrinsic activity retention, oxidation resistance, surface passivation, metal leaching, and CO tolerance under defined electrochemical and mass-transport conditions. However, RDE-based stability should not be directly equated with device-level durability. In practical flow-cell configurations and membrane–electrode assemblies (MEAs), degradation can be further influenced by catalyst-layer thickness, ionomer coverage, gas transport limitations, local water activity, carbonate accumulation, current-density distribution, electrode flooding or drying, and interfacial contact resistance. These factors become particularly important under high current densities and high ionomer contents, where accessible active sites and apparent HOR kinetics may differ significantly from those measured on RDE electrodes. Therefore, RDE durability tests should be regarded as an initial screening platform, whereas device-relevant validation under flow-cell or MEA conditions is still required to assess the practical stability of bimetallic and multimetallic alkaline HOR catalysts. Together, combining RDE-based diagnostics with device-relevant validation will help distinguish intrinsic catalyst stability from apparent retention governed by transport, catalyst-layer, or surface-area effects.

## 3 Case Study

In the design of alkaline HOR catalysts, increasing compositional complexity has been widely recognized as an effective strategy for overcoming the intrinsic adsorption limitations of monometallic surfaces. Compared with single-metal catalysts, bimetallic and multimetallic alloy systems offer a versatile platform for systematically tuning electronic structures, adsorption behaviors, and interfacial properties. In this chapter, we further discuss how the rational design of bimetallic and multimetallic alloys can be leveraged to enhance catalytic activity and durability.

### 3.1 Bimetallic Alloys

Upon incorporation of a second metal, bimetallic alloys give rise to multiple synergistic effects associated with heterogeneous elemental distributions and local coordination environments.^25,32^ These effects are generally described in terms of bifunctional, ligand, ensemble, and strain effects, all of which collectively reshape the adsorption energy landscape of catalytic sites and enable more balanced regulation of H* and OH* interactions.^24,34^ When dissimilar elements are positioned in close proximity, catalytic optimization is no longer restricted to tuning a single adsorption descriptor. Instead, the reaction pathway can be reorganized through site specialization and spatial cooperativity, thereby mitigating the kinetic limitations of HOR in alkaline media.^34,56^ Beyond the conventional bifunctional-site concept, bimetallic alloys may also alter the interfacial microenvironment by redistributing surface charge and modifying local coordination structures. These changes can affect the local electric field and the orientation of interfacial water molecules at the electrode–electrolyte boundary.^45,46^ Consequently, the influence of alloying extends beyond conventional thermodynamic descriptors and may further regulate EDL structure and proton-transport behavior.^19^

Based on compositional features and design rationale, bimetallic systems discussed in this section are categorized into three classes. Noble-metal bimetallic alloys primarily capitalize on intrinsic chemical stability and are designed to optimize electronic and site cooperativity while maintaining durability.^32,37^ Mixed noble-metal/non-noble-metal bimetallic alloys introduce oxophilic or electronically active non-noble components to accelerate interfacial charge-transfer steps, improve poisoning tolerance, and reduce noble-metal loading.^17,56,58^ In contrast, non-noble-metal bimetallic alloys are largely driven by cost and scalability considerations, aiming to replace platinum-group-metal (PGMs) catalysts through electronic compensation and interfacial engineering between nonprecious elements.^7,28^

#### 3.1.1 Noble-Metal Bimetallic Alloys

Noble-metal bimetallic alloys provide a highly controllable and chemically robust compositional space for catalyst design. Because they are composed exclusively of platinum-group metals, these systems enable clearer elucidation of intrinsic metal–metal synergistic effects while largely avoiding complications commonly associated with nonprecious components, such as dissolution, passivation, and irreversible oxidation. As a result, noble-metal bimetallic alloys serve as an important foundation for establishing design benchmarks for alkaline HOR activity and durability.

In a recent systematic study, our group established a descriptor-guided screening framework for a noble-metal bimetallic catalyst family by integrating DFT with a fine-tuned machine-learning interatomic potential (MLIP), enabling the identification of compositions with optimal alkaline HOR activity.^87^ To minimize facet- and morphology-induced variability and enable meaningful comparisons across compositions, the authors used {100}-faceted Pd nanocubes as seeds to epitaxially grow ultrathin FCC bimetallic shells with a consistent square atomic arrangement. Figure 10 outlines a descriptor-guided strategy for bimetallic catalyst design in alkaline HOR. As summarized in Figure 10A, the screening framework compares the relative Fermi levels and H_2_/OH* adsorption energies of Ir, Pt, Ru, Rh, and Au surfaces. The analysis reveals that Ir and Pt possess stronger electron-accepting tendencies, whereas Ru exhibits the strongest OH* affinity. These descriptor trends are subsequently integrated into an activity map for monometallic and bimetallic systems, highlighting the potential complementarity between metals with distinct adsorption and electronic characteristics. On this basis, Figure 10B proposes a predicted HOR activity ranking, in which RuIr and PtRu emerge as the most promising bimetallic combinations because they best combine favorable electron acceptance, H_2_ adsorption, and OH* adsorption. This descriptor-based prediction is further supported by the electrochemical results in Figure 10C,D, where RuIr catalysts exhibit the highest measured activity among the screened bimetallic compositions.

**Figure 10:**
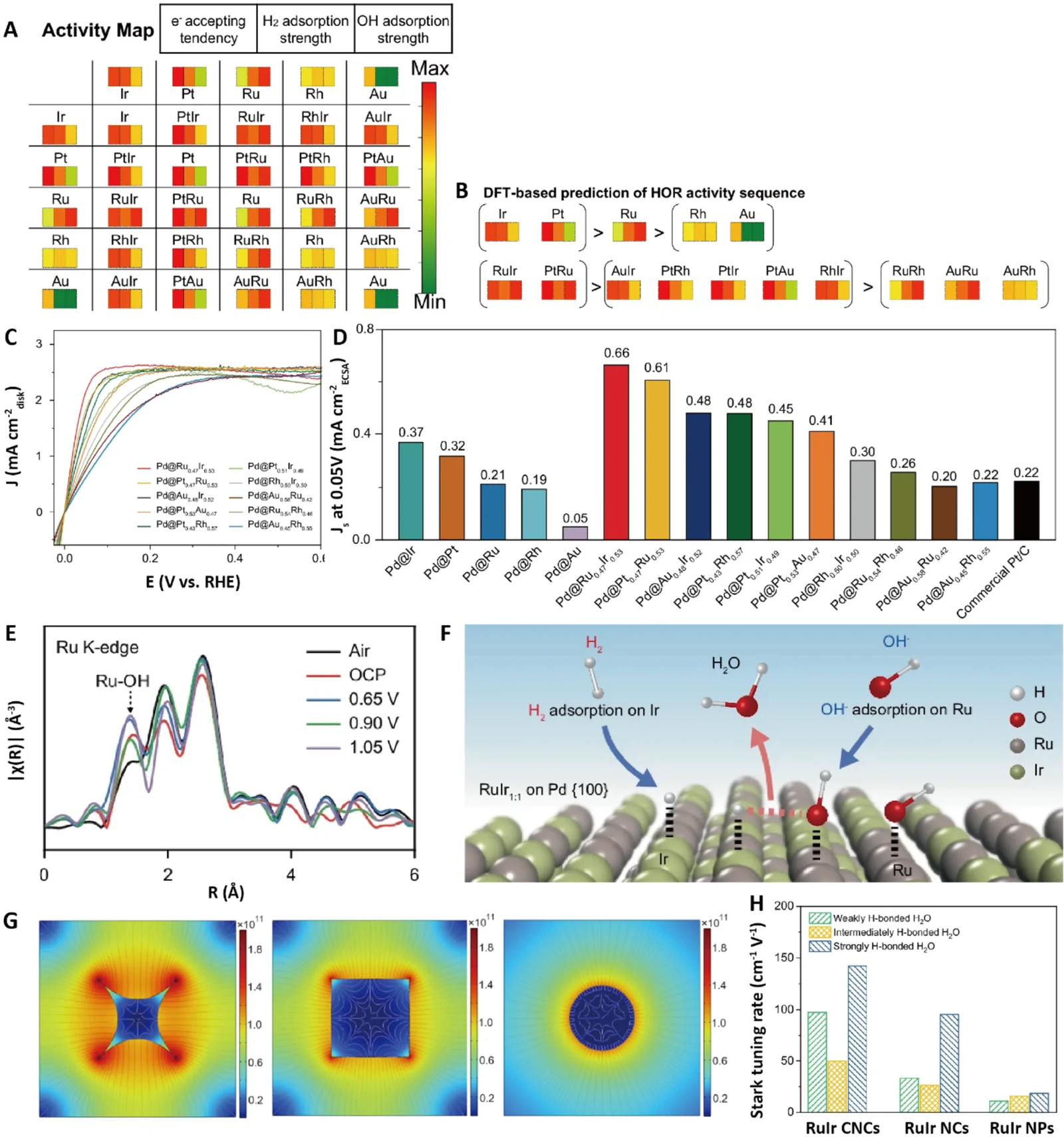
DFT-guided catalyst screening, HOR performance, and interfacial effects in RuIr bimetallic catalysts. (A) Activity map summarizing the electron accepting tendencies, H_2_ adsorption strengths, and OH adsorption strengths of different PGM metals and their bimetallic combinations based on DFT calculations. (B) Prediction of the alkaline HOR activity sequence for single metals and bimetallic alloys. (C) HOR polarization curves of Pd@bimetallic alloy core–shell nanocubes measured in H_2_-saturated 0.1 M KOH. (D) Comparison of the specific activities evaluated at 0.05 V vs RHE. (E) *Operando* FT-EXAFS spectra of Ru K-edge collected under air, OCP, and applied potentials during alkaline HOR. (F) Schematic illustration of the proposed alkaline HOR mechanism on the Pd@Ru_0.47_Ir_0.53_ surface.^87^ Adapted from ref 87 under a Creative Commons Attribution-NonCommercial-NoDerivatives 4.0 International License. (G) Simulated local electric field distributions on the surfaces of RuIr CNCs, RuIr NCs, and RuIr NPs. (H) The Stark tuning rates of interfacial water on RuIr CNCs, RuIr NCs, and RuIr NPs derived from *operando* ATR-SEIRAS spectra.^88^ Adapted from ref 88 under a Creative Commons Attribution-NonCommercial-NoDerivatives 4.0 International License.

In electrochemical testing in H_2_-saturated 0.1 M KOH, Pd@Ru_0.47_Ir_0.53_ delivered a high specific activity (*J*_s_) of 0.66 mA cm_ECSA_^–2^ at 0.05 V vs RHE, outperforming commercial Pt/C (0.22 mA cm_ECSA_^–2^) as well as other candidates in the same bimetallic series. In addition, Pd@Ru_0.47_Ir_0.53_ maintained exceptional structural integrity and retained the majority of its initial current density after ADT, whereas Pt/C exhibited pronounced performance decay. Mechanistically, *operando* XAS and theoretical analysis revealed a pronounced bifunctional synergy in RuIr. The Ru FT-EXAFS spectra collected under HOR-relevant potentials showed potential-dependent changes in the Ru local coordination environment, indicating that Ru sites dynamically interact with oxygenated species during alkaline HOR. This *operando* coordination response supports the role of Ru as an OH-affinitive component, while Ir contributes favorable H_2_ adsorption and electron-accepting characteristics. Therefore, the enhanced activity of Pd@Ru_0.47_Ir_0.53_ is not simply attributed to an averaged alloy electronic effect, but to spatially coupled Ru–Ir ensembles that balance hydrogen-related and hydroxyl-related interfacial steps. This site-specific bifunctional role is illustrated by the *operando* Ru K-edge spectra and schematic model in Figure 10E,F. Importantly, the design implication transcends the mere coexistence of dual sites, but that controlled atomic-scale mixing and site adjacency enable a more balanced H*/OH* interaction landscape, which can help mitigate deactivation risks associated with overly strong intermediate binding.

Beyond the bifunctional paradigm, emerging evidence indicates that bimetallic alloys can further regulate the interfacial microenvironment. In RuIr systems with nearly identical composition and electronic structure, geometric differences alone can substantially reshape the local electric field and interfacial water organization, thereby modulating hydrogen-bond network connectivity and proton-coupled electron transfer (PCET) kinetics. For example, Li et al. employed curvature engineering to construct RuIr nanostructures with tunable surface curvature, establishing a direct correlation between geometric effects and interfacial water structure.^88^ In H_2_-saturated 0.1 M KOH, high-curvature RuIr concave nanocages (CNCs) achieved a geometric current density of 2.62 mA cm_geo_^–2^ at 50 mV vs RHE, exceeding RuIr NCs (2.29 mA cm_geo_^–2^) and commercial Pt/C (1.46 mA cm_geo_^–2^). As shown in Figure 10G,H, these geometric effects are accompanied by curvature-dependent local electric-field distributions and Stark tuning rates that reflect distinct interfacial water structures across the RuIr nanostructures. Simulations further suggested that curvature-amplified local electric fields drive a 5.4-fold enrichment of OH_ad_ while reducing H_ad_ coverage by 79.1%. *Operando* ATR-SEIRAS combined with AIMD confirmed that such curvature stabilizes an O-down interfacial water alignment and strengthened hydrogen-bond networks that promote proton transport dynamics. This work therefore expands the design logic of bimetallic HOR catalysts from conventional bifunctional and electronic effects to explicit interfacial microenvironment engineering.

#### 3.1.2 Mixed Noble and Non-Noble Bimetallic Alloys

The central design principle of mixed noble-metal/non-noble-metal bimetallic alloy systems is to exploit the synergy between ligand effects and bifunctional interactions to coregulate intermediate adsorption and interfacial charge-transfer steps. By incorporating non-noble metals with oxophilic and multivalent characteristics, these catalysts can be engineered not only to tune hydrogen-related adsorption behavior but also to directly modulate hydroxide-involving interfacial processes in alkaline media, thereby alleviating the intrinsic kinetic limitations of monometallic noble-metal catalysts in HOR. Within this design space, IrMo alloys have emerged as an effective platform for optimizing the kinetics associated with interfacial water and hydroxide-related species. In particular, the oxophilicity of Mo, together with alloying-induced electronic modulation, reshapes the adsorption-energy landscape and promotes more favorable H_2_O/OH-related interfacial processes, leading to enhanced alkaline HOR performance.^89^

In H_2_-saturated 0.1 M KOH electrolyte, IrMo_0.59_ exhibited the best HOR performance among the evaluated compositions. Its kinetic current density (*j*_k,m_) normalized to noble-metal mass reached 0.50 mA μg_PGM_^–1^, while its exchange current density (*j*_0,s_) normalized to ECSA reached 1.15 mA cm_PGM_^–1^. Both metrics are approximately four times higher than those of commercial Pt/C and pure Ir, indicating a substantial improvement in intrinsic kinetics upon Mo incorporation. As shown in Figure 11A–C, IrMo_0.59_ exhibits higher HOR current densities, kinetic current response, and *j*_0,s_ than Ir nanoparticles and Pt/C. In terms of durability, IrMo_0.59_ showed negligible loss of HOR activity after 1000 accelerated potential cycles while maintaining stable morphology, crystal phase, and surface chemical states under the tested alkaline conditions. Notably, the HOR polarization curve of IrMo exhibited a pronounced current decrease in the high-potential region, which is attributed to its relatively strong oxygen affinity. Such strong oxygen affinity can reduce the number of available hydrogen adsorption sites and thus lower intermediate turnover efficiency. This result highlights that catalyst design must balance the promotion of hydroxyl-related interfacial steps against excessive oxophilicity that may induce site blocking, in order to simultaneously achieve high activity and stability. DFT calculations further clarified the origin of this synergistic effect. The corresponding structural model, calculated reaction energetics, and proposed reaction pathway are shown in Figure 11D–F. On the IrMo surface, Mo sites are preferentially occupied by H_2_O, which effectively optimizes the energetics of H_2_O and OH* and thereby facilitates the Volmer step in alkaline HOR. Importantly, if hydrogen binding energy is used as the sole activity descriptor, IrMo­(H_2_O) would be predicted to exhibit poorer HOR activity because of its more negative Δ*G*_H*_. However, experimental results show that IrMo performs significantly better than such a prediction. This discrepancy indicates that the performance enhancement arises primarily from more favorable hydroxyl adsorption and a facilitated Volmer step, rather than from simple optimization of H* adsorption strength alone.

**Figure 11:**
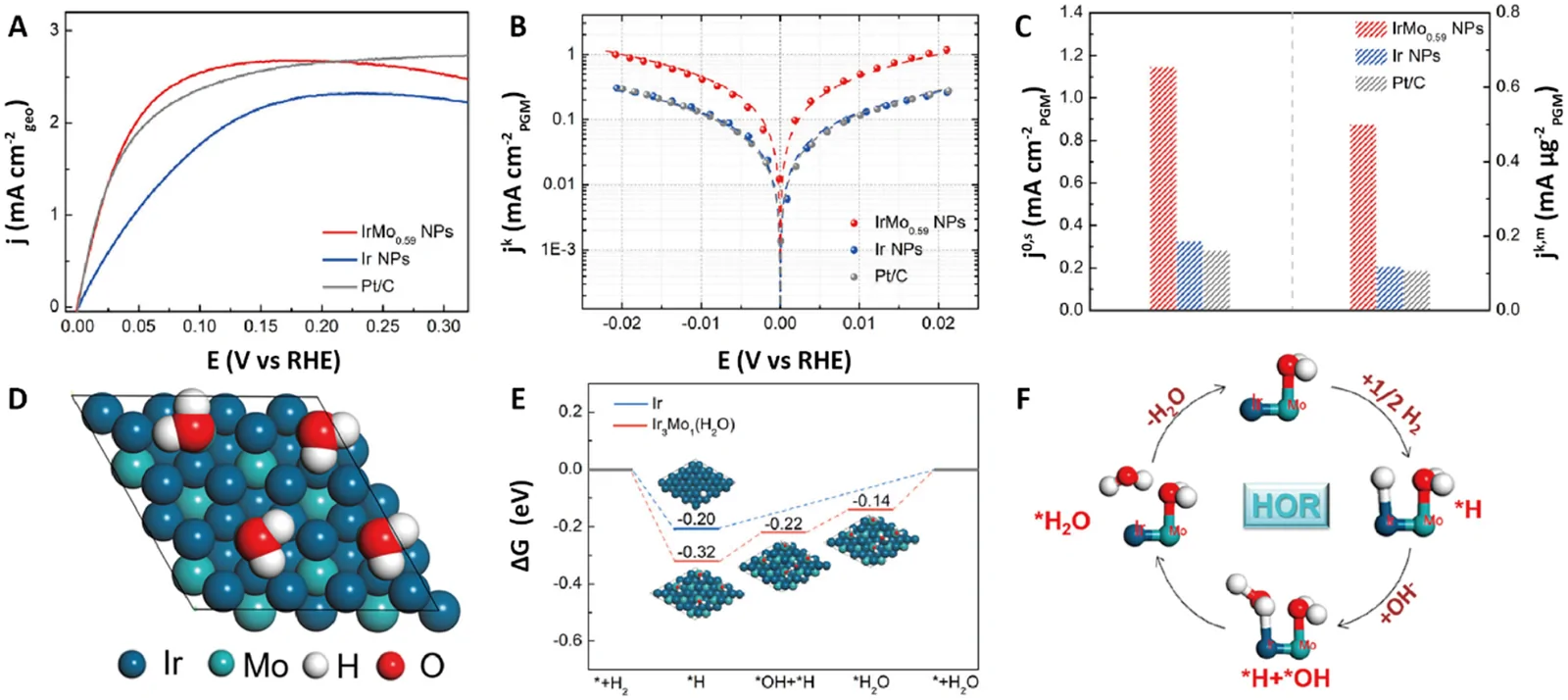
HOR performance, reaction energetics, and proposed reaction pathway of IrMo_0.59_nanoparticles. (A) HOR polarization curves of IrMo_0.59_ NPs, Ir NPs, and Pt/C in H_2_-saturated 0.1 M KOH. (B) Corresponding Tafel plots derived from the ECSA-normalized kinetic current densities. (C) Comparison of the exchange current densities and mass activities evaluated at 10 mV vs RHE. (D) Optimized structural model of Ir_3_Mo_1_(H_2_O). (E) Gibbs free-energy diagram of HOR reaction intermediates on Ir and Ir_3_Mo_1_(H_2_O). (F) Schematic illustration of the alkaline HOR mechanism on Ir_3_Mo_1_(H_2_O).^89^ Adapted with permission from ref 89. Copyright 2020 American Chemical Society.

#### 3.1.3 Non-Noble-Metal Bimetallic Alloys

Non-noble-metal bimetallic alloys are designed to deliver practically relevant alkaline HOR activity while maximizing cost effectiveness, although their performance is often limited by surface passivation under anodic polarization.^90–92^ Among nonprecious candidates, Ni-based alloys have served as a particularly important platform because Ni is one of the few non-noble metals that can exhibit measurable HOR activity in alkaline media. However, pristine Ni is generally constrained by excessively strong H* binding and is susceptible to oxidation-induced deactivation at elevated potentials.^90,93^ In this context, Ni–Mo bimetallics represent a prototypical design strategy. Incorporation of the more oxophilic Mo component can simultaneously weaken overly strong H* adsorption on Ni sites while increasing the availability of OH* species for interfacial reactions, thereby pushing Ni-based systems closer to noble-metal benchmarks in the low-overpotential regime.^92,94^

In H_2_-saturated 0.1 M KOH at 1600 rpm, Ni_4_Mo alloy nanoparticles delivered an anodic geometric current density of 2.2 mA cm^–2^ at 50 mV versus RHE, exceeding that of Pt/C (1.8 mA cm^–2^) and approaching that of PtRu/C (2.6 mA cm^–2^) under identical conditions. Notably, the HOR activity of Ni_4_Mo began to decline at anodic potentials above approximately 0.2 V versus RHE, which is attributed to surface oxidation of Ni. This behavior highlights that the practical operating window of non-noble catalysts remains restricted by passivation under oxidative conditions. Importantly, during constant-potential stability testing at 50 mV versus RHE, Ni_4_Mo maintained a stable current density of approximately 2.2 mA cm^–2^ throughout the measurement, in contrast to the gradual activity decay observed for Pt/C and PtRu/C. These results suggest that Ni_4_Mo can achieve a favorable balance between activity and stability within the low-potential alkaline HOR regime. As illustrated in Figure 12A,B, Ni_4_Mo exhibits substantially higher HOR activity in the low-overpotential region than that of pristine Ni, while maintaining excellent stability during chronoamperometric testing.

**Figure 12:**
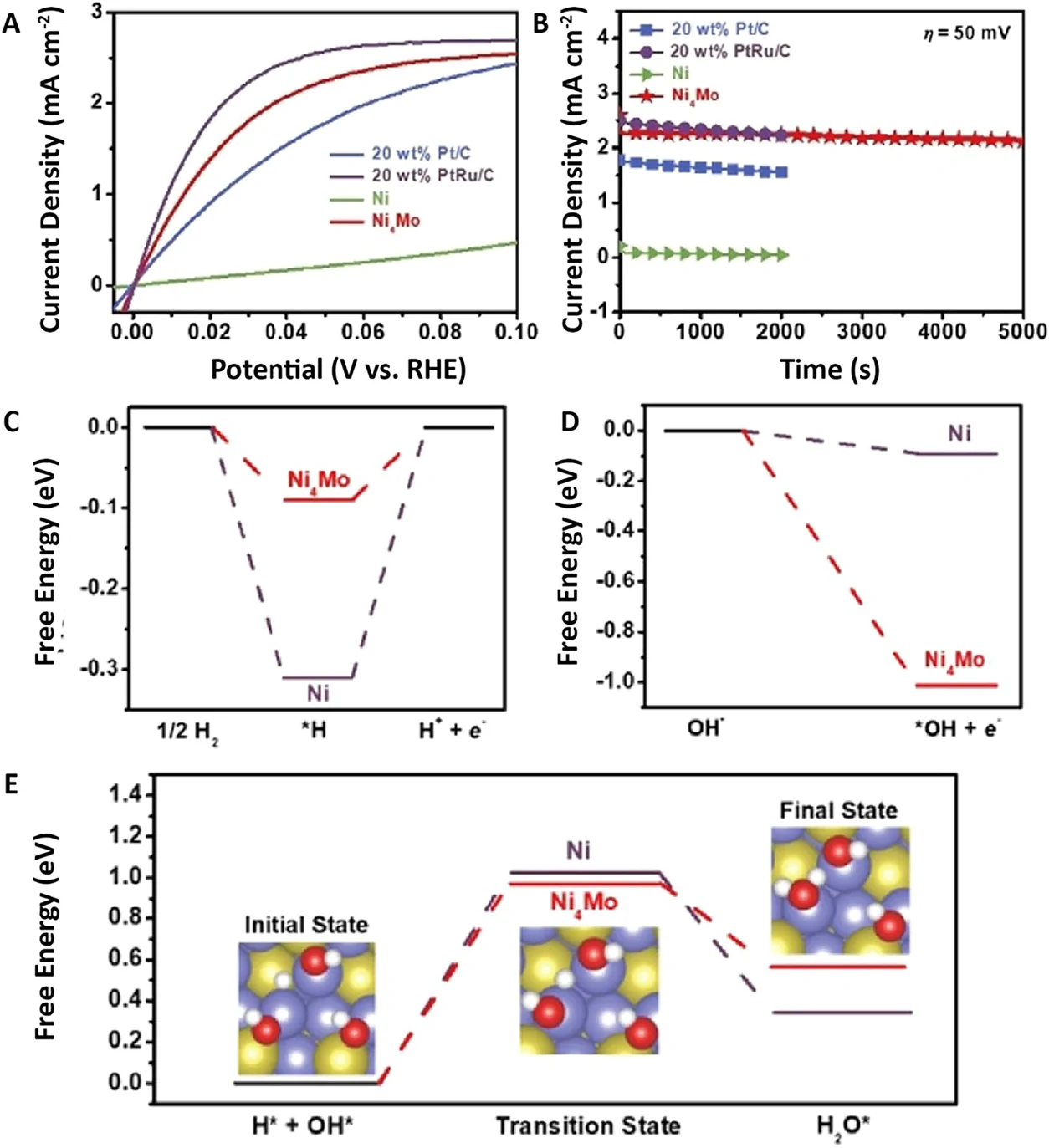
Alkaline HOR performance and calculated reaction energetics of Ni_4_Mo. (A) HOR polarization curves of Ni_4_Mo, Ni, 20 wt % Pt/C, and 20 wt % PtRu/C in H_2_-saturated 0.1 M KOH. (B) Chronoamperometric current responses of Ni_4_Mo, Ni, 20 wt % Pt/C, and 20 wt % PtRu/C measured at 50 mV. (C, D) Gibbs free-energy diagrams for H* and OH* adsorption on Ni_4_Mo­(211) and Ni(111). (E) Calculated energy barrier for water formation on Ni_4_Mo and Ni, with the initial, key transition, and final states illustrated in the insets.^94^ Adapted with permission from ref 94. Copyright 2021 Wiley-VCH GmbH.

Mechanistically, DFT analysis provides a consistent explanation for the observed activity enhancement. The corresponding adsorption energetics and reaction barriers are summarized in Figure 12C–E. Mo incorporation shifts hydrogen binding toward a more thermoneutral regime, with a predicted Δ*G*_H*_ of −0.09 eV on Ni_4_Mo­(211), compared with −0.30 eV on Ni(111), indicating effective mitigation of excessively strong H* adsorption on Ni. At the same time, OH* preferentially adsorbs on the more oxophilic Mo sites, suggesting that increased OH* availability can facilitate alkaline interfacial reaction steps. Beyond thermodynamic considerations, CI-NEB calculations further showed that Mo lowers the energy barrier for the water-formation step, H* + OH* → H_2_O, from 1.03 eV on Ni to 0.95 eV on Ni_4_Mo. Taken together, this case illustrates that the design of non-noble-metal bimetallic catalysts is most effective when H* adsorption, OH* adsorption, and the key interfacial reaction barrier are co-optimized simultaneously. At the same time, further stability-oriented engineering remains necessary to expand the oxidation-limited operating window under practical anodic conditions.

### 3.2 Trimetallic Alloys

Compared with bimetallic alloys, trimetallic systems introduce an additional compositional degree of freedom that markedly expands the design space for tuning alkaline HOR kinetics.^25,28^ The purpose is not merely to add a third element, but to exploit ternary electronic interactions and multisite synergy to better balance the coupled adsorption requirements that govern alkaline HOR.^28^ This expanded compositional space can help alleviate the adsorption trade-offs frequently encountered in bimetallic alloys, because hydrogen- and hydroxyl-related intermediates often compete for overlapping surface ensembles, and their coverages can vary substantially with potential and electrolyte conditions.^24,32^ In addition, trimetallic formulations are often used as a structural lever. By modifying local coordination statistics and lattice distortion, they can broaden the distribution of adsorption strengths and help mitigate operando constraints such as site blocking, potential-induced surface restructuring, and passivation.^28^

Among the many possible trimetallic design routes, this section focuses on two representative strategies. The first is an implantation strategy, in which a highly active bimetallic framework, such as PtRu, serves as the backbone while a third component with pronounced oxophilic character is introduced in a controlled manner.^95^ The goal is to increase the density of oxophilic motifs without disrupting the primary framework, thereby promoting hydroxyl-related interfacial steps and improving robustness against potential-dependent surface-state changes. The second is a Sabatier-principle-guided alloying strategy, which combines PGM elements with additional metals located on opposite sides of the adsorption-strength spectrum.^96^ By adjusting atomic ratios and overall composition, this approach seeks to tune the adsorption-strength distribution of key intermediates toward a near-optimal window, as commonly described by adsorption volcano plots, thus enabling high catalytic performance at reduced precious-metal content.^52,96^

#### 3.2.1 Oxophilic-Element-Implanted Trimetallic Alloys

In the design of alkaline HOR catalysts, PtRu alloys are widely recognized as one of the most competitive bimetallic platforms because they can partially balance the interfacial requirements for hydrogen- and hydroxyl-related steps.^95^ However, their performance remains constrained by the intrinsically coupled adsorption demands of the reaction interface. Pt sites often retain hydrogen-related species too strongly, which can hinder reaction kinetics, whereas Ru-derived oxophilic motifs are needed to facilitate OH-related interfacial processes under alkaline conditions. This delicate balance limits the extent to which the PtRu framework alone can be optimized. To address this limitation, recent work has adopted PtRu nanowires as a high-intrinsic-activity scaffold and introduced a third functional metal through an implantation strategy, thereby constructing i-M-PR trimetallic systems (M = Mn, Fe, Co, Ni, Cu, Zn, or Ga). The synthesis concept, nanowire morphology, and structural characteristics of the implanted PtRu framework are shown in Figure 13A–D. Rather than simply altering the overall composition, this strategy is designed to tailor the local interfacial microenvironment around the PtRu backbone. The implanted element perturbs the electronic structure and local coordination environment of neighboring PtRu sites, thereby reshaping the adsorption-energy distribution associated with H- and OH-involving elementary steps. As a result, the trimetallic framework provides a more flexible platform for regulating interfacial kinetics than the parent PtRu alloy alone.

**Figure 13:**
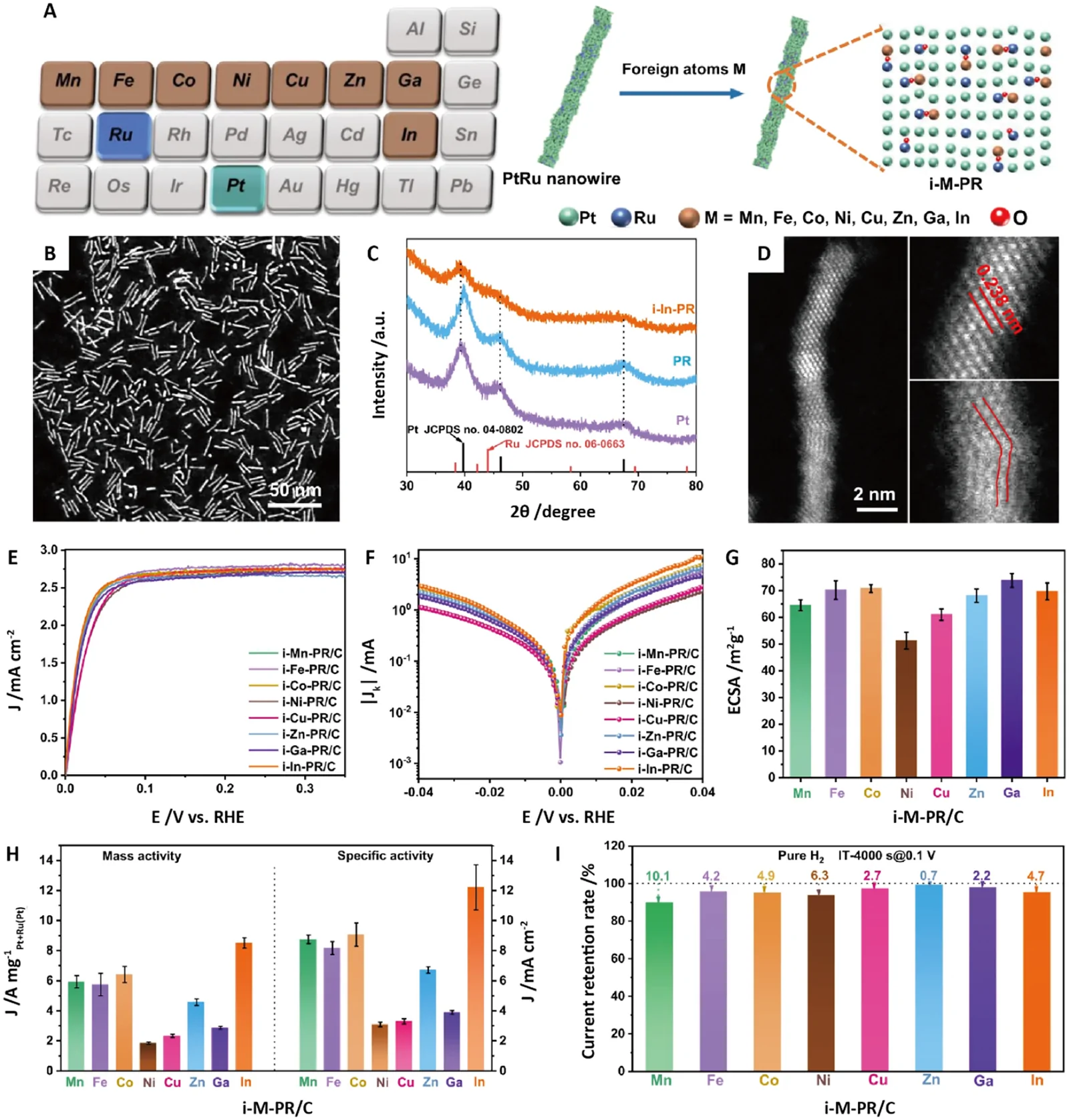
Incorporation of foreign atoms into PtRu nanowires and the corresponding HOR performance of i-M-PR/C catalysts. (A) Schematic illustration of the foreign atoms incorporation strategy into PtRu nanowires. (B) High-angle annular dark-field scanning transmission electron microscopy (HAADF-STEM) image of the nanowires. (C) XRD patterns of Pt, PR, and i-In-PR. (D) Aberration-corrected HAADF-STEM images of an individual nanowire. (E) HOR polarization curves of the i-M-PR/C catalysts measured in H_2_-saturated 0.1 M KOH. (F) Corresponding Tafel plots derived from the kinetic current densities. (G) Comparison of the ECSAs of the i-M-PR/C at 50 mV vs RHE. (H) ECSA-normalized specific activities and mass activities at 50 mV vs RHE. (I) Current retention rate of the catalysts measured in H_2_-saturated 0.1 M KOH at 100 mV vs RHE.^95^ Adapted from ref 95 under a Creative Commons Attribution 4.0 International License.

To quantify the role of the implanted element, HOR activity is evaluated in H_2_-saturated 0.1 M KOH using polarization measurements, with the mass activity at 50 mV vs RHE chosen as a representative metric for the low-overpotential regime. Across a dopant library, the polarization responses and kinetic-current trends vary systematically with the implanted element, indicating an element-dependent performance fingerprint (Figure 13E,F). These activity differences are further reflected in the corresponding mass and specific activity comparisons shown in Figure 13H. The dopant content is first optimized, yielding an optimum value of 3.9%; other implanted metals are subsequently controlled at comparable contents to enable a consistent head-to-head comparison across dopants. Under these conditions, i-In-PR/C delivered a mass activity of 8.51 A mg_(Pt + Ru)_^–1^ and a specific activity of 12.21 mA cm^–2^, indicating that implantation of a single oxophilic metal can markedly enhance PtRu-catalyzed HOR in the low-overpotential region. Notably, the ECSA across i-M-PR/C does not show a commensurate enlargement (Figure 13G), supporting that the activity gain is primarily associated with modified site properties rather than a simple surface-area effect. In addition to activity, element-dependent stability signatures are observed. For instance, i-Zn-PR/C exhibited the minimal decay during a constant-potential hold at 100 mV vs RHE, retaining 99.3% of its initial current after 4000 s, suggesting that implantation can significantly improve operational robustness. The element-dependent stability trends across the various implanted catalysts are summarized in Figure 13I.

Mechanistically, the beneficial effect of implantation is attributed to coordinated electronic modulation of the PtRu backbone. The implanted element shifts the Pt d-band center downward, weakening the adsorption of H* and CO, while simultaneously tuning the electronic state of Ru sites to promote OH* adsorption. Such concerted regulation helps better align hydrogen-related elementary steps with hydroxyl-mediated interfacial processes, thereby relieving the adsorption trade-off that often limits bimetallic catalysts. This interpretation is further supported by computed energetics, which reveal a more favorable HOR free energy landscape and a more accessible CO oxidation pathway for the implanted trimetallic system. Interestingly, building on the observation that different implanted elements can contribute complementary benefits, the concept is further extended through coimplantation. With Zn and In introduced together, i-ZnIn-PR/C shows a stronger low-overpotential polarization response and achieves a mass activity of 10.2 A mg_(Pt + Ru)_^–1^ at 50 mV vs RHE, substantially exceeding Pt/C at 0.27 A mg_Pt_^–1^ and PtRu/C at 1.24 A mg_(Pt + Ru)_^–1^. It also shows only a 5.3% loss over 10,000 s in a constant-potential test and maintains 84.9% of its initial current after 5000 s in a CO-tolerance evaluation. Collectively, these results highlight how implantation on a PtRu framework can be used as a tunable design handle to balance activity, stability, and poisoning tolerance, and how a trimetallic implantation concept can be naturally extended toward multielement synergy when complementary effects are identified.

#### 3.2.2 Adsorption-Balanced Trimetallic Alloys

Beyond implantation strategies, another important direction in trimetallic alloy design is deliberate compositional regulation guided by the Sabatier principle. The central concept is to incorporate both relatively strong-binding and weak-binding components within a single alloy lattice, thereby alleviating the adsorption-energy trade-offs among key reaction intermediates. In this framework, our group used Ir as the principal backbone and simultaneously alloyed it with Mo as a relatively strong-binding component and Cu as a relatively weak-binding component.^96^ Through ternary alloying and compositional balancing, this strategy enables the adsorption energetics of H*- and OH*-related species to be tuned toward a more favorable window, while keeping the Ir content below 20 atom %. For alkaline HOR evaluation, Ir_0.2_Mo_0.6_Cu_0.2_, Ir_0.2_Mo_0.4_Cu_0.4_, and Ir_0.2_Mo_0.2_Cu_0.6_ with different Mo/Cu ratios are tested in H_2_-saturated 0.1 M KOH, with mass activity at low overpotentials used as the key metric. After normalizing the current to noble-metal mass, Ir_0.2_Mo_0.4_Cu_0.4_ delivered 0.90 and 1.54 A mg_PGM_^–1^ at 0.05 and 0.10 V vs RHE, respectively (Figure 14A,B). In accelerated durability testing over 9000 CV cycles, commercial Pt/C exhibited activity losses of 38 and 28% at 0.05 and 0.10 V vs RHE, whereas Ir_0.2_Mo_0.4_Cu_0.4_ decreased by only 21 and 7%, suggesting improved durability even at low PGM content. This durability advantage is summarized in Figure 14C,D.

**Figure 14:**
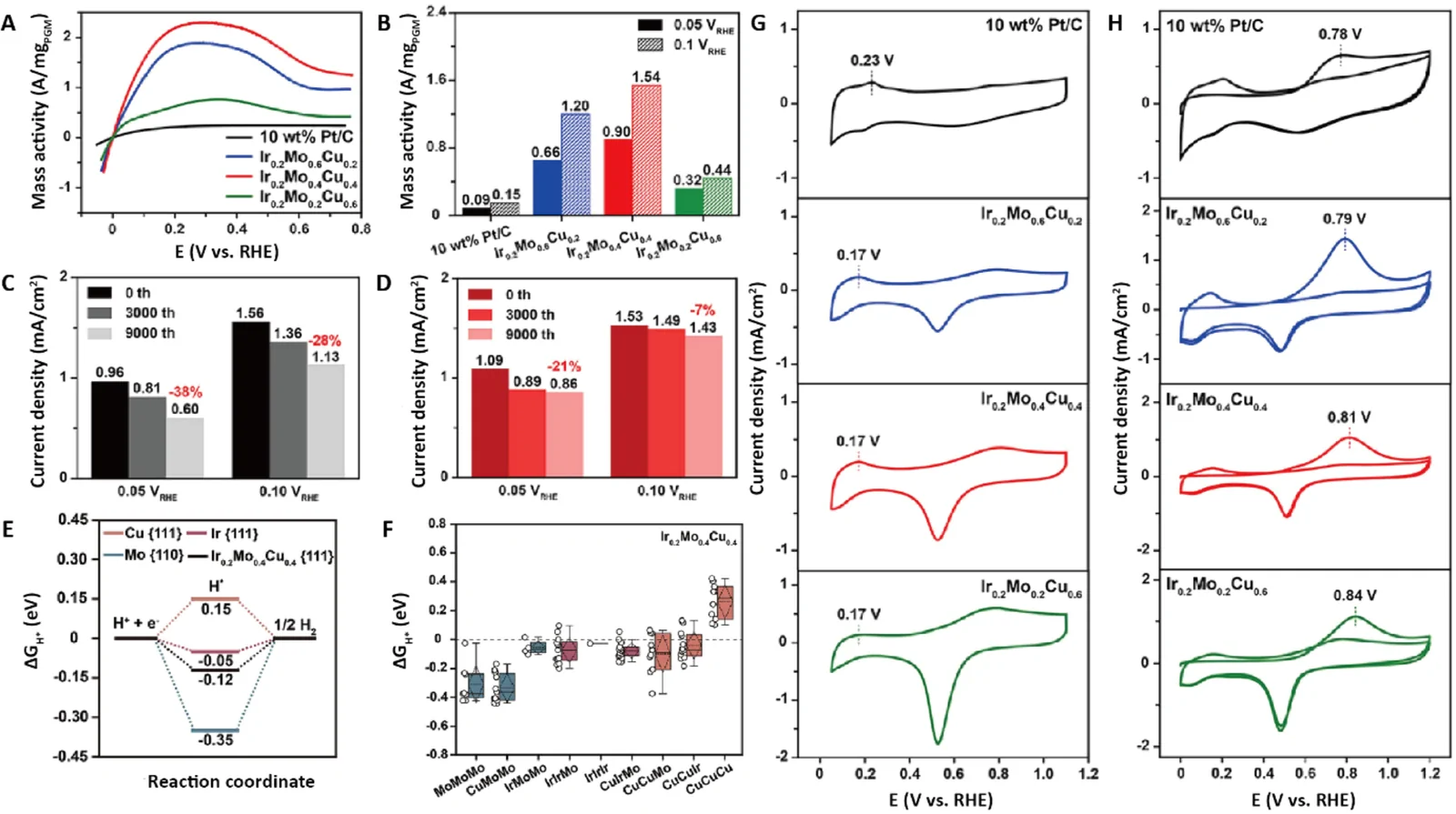
Composition-dependent HOR activity, stability, and adsorption free energy of Ir–Mo–Cu catalysts. (A, B) Comparison of the mass activities of commercial Pt/C, Ir_0.2_Mo_0.6_Cu_0.2_, Ir_0.2_Mo_0.4_Cu_0.4_, and Ir_0.2_Mo_0.2_Cu_0.6_, with the corresponding values evaluated at 0.05 and 0.1 V vs RHE. (C, D) HOR polarization curves of Pt/C and Ir_0.2_Mo_0.4_Cu_0.4_ recorded before and after 9000 CV cycles. (E) Calculated reaction-coordinate diagram for H* adsorption on Ir, Mo, Cu, and Ir_0.2_Mo_0.4_Cu_0.4_. (F) Comparison of the Δ*G*_H*_ values at hollow sites on different metal surfaces. (G) Comparison of the CV of Ir_0.2_Mo_*x*_Cu_0.8‑*x*_ with different Mo/Cu ratios measured in N_2_ saturated 0.1 M KOH for H_upd_ analysis. (H) CO stripping voltammograms of various catalysts, highlighting differences in surface OH* binding strength and CO oxidation characteristics.^96^ Adapted with permission from ref 96. Copyright 2025 Wiley-VCH GmbH.

DFT calculations are performed to evaluate Δ*G*_H*_ on representative Ir_0.2_Mo_0.4_Cu_0.4_ included as single-element reference surfaces. As expected, the calculated average Δ*G*_H*_ values for Ir, Mo, and Cu are −0.05, −0.35, and 0.15 eV, respectively, corresponding to moderate, strong, and weak hydrogen-binding characteristics. To account for the compositional complexity of the Ir_0.2_Mo_0.4_Cu_0.4_ surface, more than 100 hydrogen-adsorbed configurations are sampled to capture the diversity of local adsorption environments. As shown in Figure 14E, Ir_0.2_Mo_0.4_Cu_0.4_ exhibited an average Δ*G*_H*_ of −0.12 eV, which lies within a favorable thermodynamic window for hydrogen adsorption, indicating binding that is neither excessively strong nor overly weak.

To connect adsorption tuning with the experimentally observed performance differences, H_upd_ and CO-stripping are employed as electrochemical probes to examine interfacial adsorption characteristics. The H_upd_ peak positions of the three compositions are similar, whereas the peak areas varied systematically with composition, consistent with different extents of H-related adsorption; notably, the H_upd_-associated signal increased with increasing Mo content. CO-stripping measurements, which reflect the availability of oxygenated surface species and the OH-involving removal of adsorbed CO*, also showed a clear compositional dependence. As the Cu content increased, the CO-stripping peak shifted progressively to higher potentials, following the sequence Pt/C (0.78 V vs RHE) < Ir_0.2_Mo_0.6_Cu_0.2_ (0.79 V vs RHE) < Ir_0.2_Mo_0.4_Cu_0.4_ (0.81 V vs RHE) < Ir_0.2_Mo_0.2_Cu_0.6_ (0.84 V vs RHE). This trend suggests that Ir_0.2_Mo_0.4_Cu_0.4_ resides in a relatively balanced interfacial adsorption regime, consistent with its superior HOR performance in the low-overpotential region. This balanced adsorption behavior is consistent with the H_upd_ and CO-stripping responses shown in Figure 14G,H. Overall, these results support that ternary alloying can fine-tune an effective adsorption window through the complementary roles of strong- and weak-binding elements. To further probe the catalytic mechanism under working conditions, *operando* XAS is performed at the Ir L_3_-edge, Mo K-edge, and Cu K-edge over a range of applied potentials during alkaline HOR. The potential-dependent spectral evolution observed for all three elements indicates dynamic electronic redistribution within the trimetallic framework. In particular, the Mo response suggests a stronger coupling to hydroxide-related interfacial adsorption, highlighting its role in regulating OH*-involving steps. Together with the electrochemical and DFT results, these operando findings reinforce the view that Sabatier-guided ternary alloying can create a more balanced adsorption environment for alkaline HOR.

### 3.3 HEAs

HEA catalysts provide a powerful platform for advancing alkaline HOR because they combine wide compositional tunability with intrinsically rich surface chemistry.^97,98^ In a strict sense, HEAs are multicomponent alloys that typically contain at least five principal elements, often designed near equiatomic ratios, which distinguishes them from conventional binary or ternary alloys and enables a much broader design space.^99^ Their catalytic promise is frequently rationalized by the four core effects, namely the high entropy effect, severe lattice distortion, sluggish diffusion, and the so-called cocktail effect.^99,100^ By confining multiple elements into a single lattice, the high entropy effect can stabilize a solid solution phase and reduce the driving force toward ordered intermetallic compounds or macroscopic phase separation, which is beneficial for maintaining a stable and well mixed surface under electrochemical operation. Meanwhile, severe lattice distortion and the associated local strain fields can be leveraged as an additional descriptor to tune adsorption energetics, because strain can shift the binding strengths of key intermediates and reshape the distribution of active ensembles.^100^ In parallel, sluggish diffusion can suppress extensive surface segregation and structural coarsening, thereby improving durability under potential cycling.^27,99^ Finally, the cocktail effect reflects the collective electronic and geometric interplay among dissimilar elements, producing a high density of distinct local coordination motifs and electronic environments that generate diverse adsorption ensembles.^98,100^ Such ensembles can be tuned to balance hydrogen and hydroxyl related interactions, which is central to alleviating the alkaline HOR kinetic penalty while retaining stability.^97,98^

In the following, we summarize two major directions that have emerged for HOR-oriented HEA design: noble-metal HEAs that prioritize chemical robustness and well-defined PGM ensemble effects, and mixed noble plus non-noble HEAs that incorporate oxophilic or electronically active components to further optimize interfacial steps while reducing precious metal usage.

#### 3.3.1 Noble-Metal HEAs

Noble-metal HEAs, typically constructed from platinum group metals, offer a comparatively stable compositional space for alkaline HOR because they largely avoid the dissolution and passivation liabilities often introduced by nonprecious components, while still enabling substantial multielement ensemble and ligand effects.^99^ In an early yet pivotal example that helped establish subsequent benchmarking and design baselines, the quinary PdPtRhIrRu HEA system is shown to deliver clear activity and durability advantages when genuine solid solution mixing is achieved.^99^ Under H_2_-saturated 0.1 M KOH, solid solution PdPtRhIrRu HEA nanocrystals (with and without dendritic morphology) exhibited higher geometric current densities at 0.1 V vs RHE (3.00 and 2.68 mA cm^–2^) than both a phase separated PdPtRhIrRu analog (2.63 mA cm^–2^) and commercial Pt/C (1.34 mA cm^–2^). As shown in Figure 15A,B, both solid-solution PdPtRhIrRu samples outperform the phase-separated counterpart and commercial Pt/C in the alkaline HOR polarization response and current density measured at 0.1 V vs RHE. Beyond the initial activity, the same controlled comparison underscored durability as a defining advantage of the solid-solution HEA concept in alkaline HOR: after 3000 accelerated cycles, the solid solution samples still retained 85.0% (2.55 mA cm^–2^) and 95.9% (2.57 mA cm^–2^) of their geometric current densities at 0.1 V vs RHE, whereas Pt/C and the phase-separated counterpart exhibited obvious deterioration (Figure 15C). The improved stability is attributed to high entropy stabilization together with multielement mixing, which suppresses severe restructuring and helps preserve morphology under electrochemical perturbation. In retrospect, the importance of this work lies not only in the performance metrics themselves, but also in the clear message that achieving genuine atomic level mixing, rather than merely combining multiple metals, is critical for realizing both the activity gain and the durability benefit in alkaline HOR.

**Figure 15:**
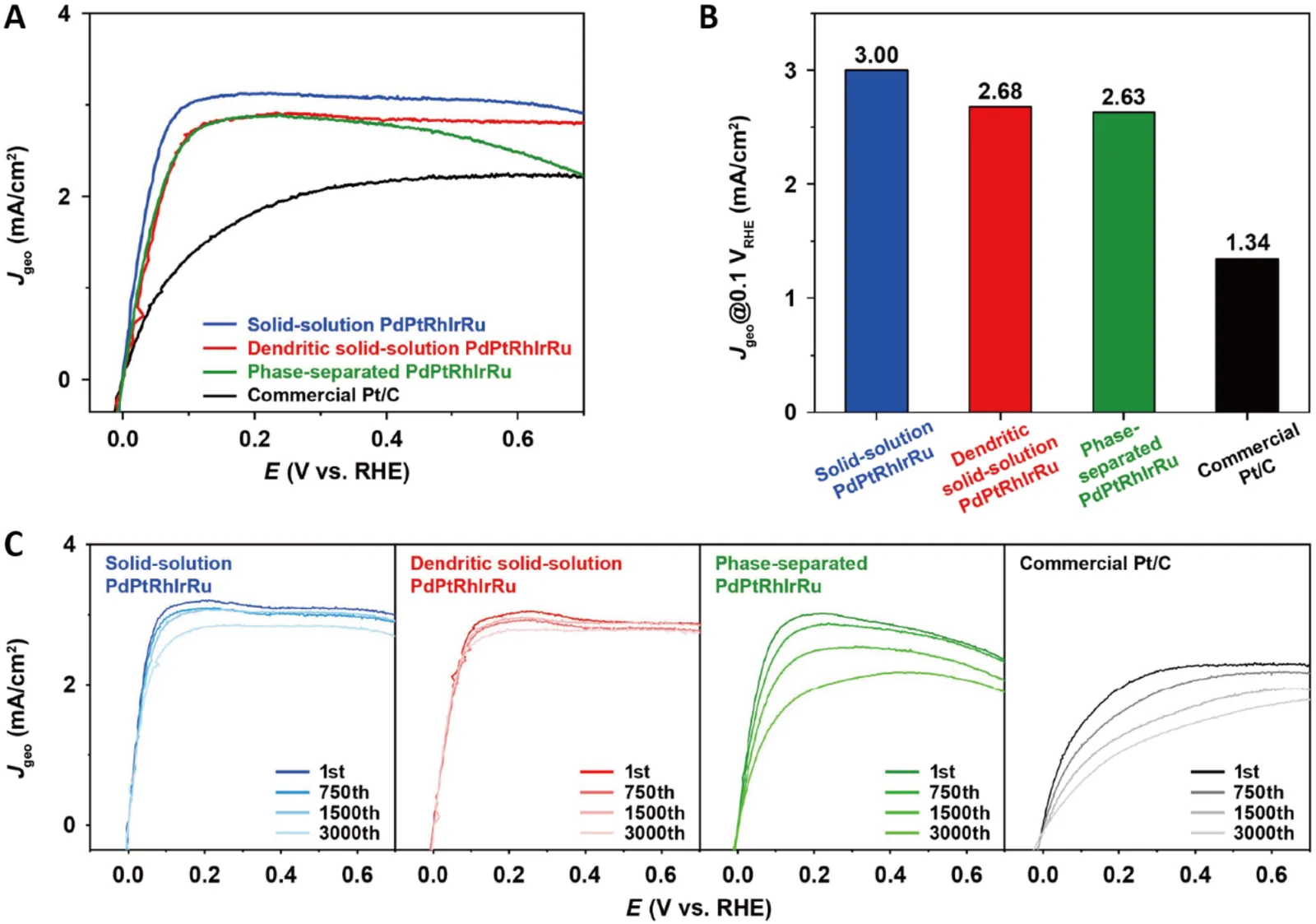
Structure-dependent HOR performance and stability of PdPtRhIrRu nanocrystals. (A) HOR polarization curves of solid-solution, dendritic solid-solution, and phase-separated PdPtRhIrRu nanocrystals, and commercial Pt/C, in H_2_-saturated 0.1 M KOH. (B) Comparison of geometric current densities evaluated at 0.1 V vs RHE. (C) Comparison of HOR polarization curves recorded before and after 1, 750, 1500, and 3000 CV cycles.^99^ Reproduced from Liu et al., Science Advances, DOI: 10.1126/sciadv.adf9931 (2023), AAAS.

#### 3.3.2 Mixed Noble and Non-Noble HEAs

Mixed noble and non-noble HEAs extend the HEA design space for alkaline HOR by combining the chemical robustness of PGMs with the functional roles of oxophilic or electronically active non- precious elements. The central concept is to decouple and simultaneously optimize the two adsorption requirements that often underpin the alkaline HOR penalty, namely weakening overly strong hydrogen adsorption while strengthening hydroxyl-related interactions that facilitate interfacial water or Volmer-type steps. In practice, this is achieved by engineering multielement surface ensembles in which PGM rich motifs provide efficient H_2_ activation and near optimal H binding, whereas non noble components introduce oxophilic sites that promote OH adsorption and interfacial charge transfer.

A representative example is the quinary PdNiRuIrRh HEA catalyst, which illustrates how mixing PGMs with an oxophilic non-noble component can simultaneously enhance low-overpotential activity, intrinsic kinetics, and robustness.^98^ Under H_2_-saturated 0.1 M KOH, the catalyst achieves a transport-corrected kinetic current density (*j*_k_, normalized to geometric area via Koutecky–Levich analysis) of 22.67 mA cm^–2^ at 50 mV vs RHE, evidencing a pronounced kinetic advantage in the low-overpotential HOR regime (Figure 16A,B). The intrinsic kinetics are further captured by an ECSA-normalized exchange current density (*j*_0,s_) of 1.19 mA cm^–2^, indicating that the enhancement is not merely a utilization effect. Consistently, the catalyst also delivers an ultrahigh mass activity (*j*_k,m_) of 3.25 mA μg^–1^ at 50 mV, corresponding to an approximately 8-fold improvement over commercial Pt/C under the same metric. Importantly, durability is explicitly benchmarked before considering impurity tolerance: after 1000 CV cycles, the HOR polarization curve of the HEA nearly coincides with the initial state, and the *j*_0,s_ shows only a 4.8% decrease, whereas the reference samples deteriorate rapidly under the same cycling protocol; the retained crystalline phase after the stability test further supports structural resilience (Figure 16C,D). Building on this cycling stability, CO tolerance is then evaluated: in H_2_ containing 100 ppm of CO, Pt/C exhibits a rapid current decay (63%), whereas the HEA shows only a modest decline, consistent with improved operational robustness under CO-related polarization. Mechanistically, machine-learning-potential Monte Carlo simulations and adsorption analyses suggest that the HEA surface is enriched in Pd and Ni and dominated by Pd–Pd–Ni and Pd–Pd–Pd hollow-FCC ensembles that approach thermoneutral Δ*G*_H*_, while Ni and Ru act as the most oxophilic centers that strengthen OH adsorption; together, these multielement ensembles are proposed to co-optimize H* adsorption–desorption and OH*-assisted interfacial steps, thereby accelerating alkaline HOR while sustaining stability. The corresponding H and OH adsorption energetics are shown in Figure 16E,F.

**Figure 16:**
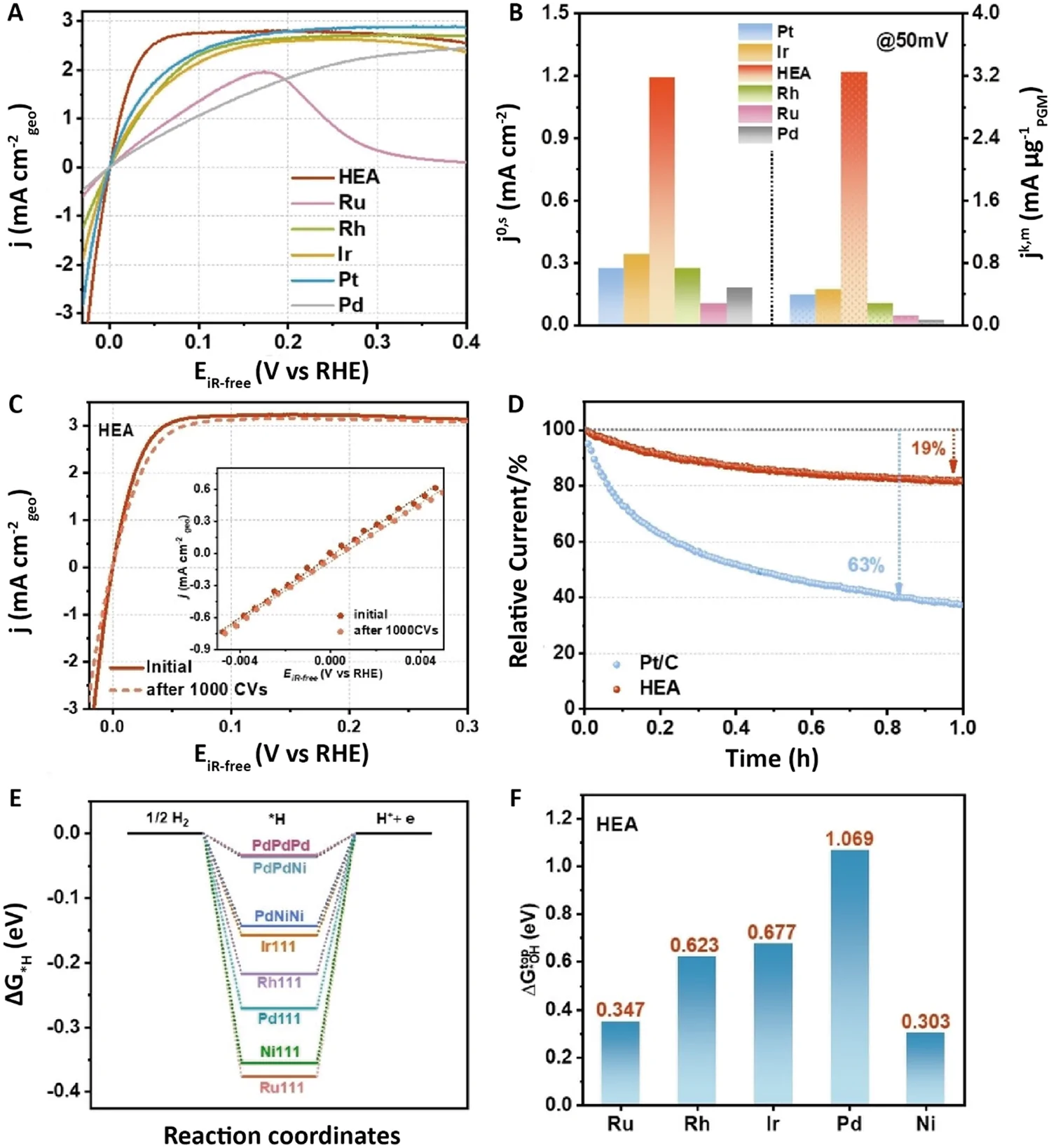
HOR performance, stability, and calculated adsorption energetics of the HEA catalyst. (A) HOR polarization curves of HEA, Pd, Ru, Ir, Rh, and commercial Pt/C in H_2_-saturated 0.1 M KOH. (B) Comparison of the ECSA-normalized exchange current densities (*j* ^0,s^) and mass activities (*j* ^k,m^) at 50 mV vs RHE. (C) HOR polarization curves of the HEA catalyst measured before and after 1000 CV cycles, with the enlarged low-potential region shown. (D) Chronoamperometric current retention of HEA and Pt/C in 0.1 M KOH saturated with 100 ppm of CO/H_2_ at 0.05 V vs RHE. (E) Calculated free-energy diagram for H* adsorption at fcc hollow sites on HEA and pure-metal (111) surfaces. (F) Calculated OH* adsorption free energies on different metal-center sites.^98^ Adapted with permission from ref 98. Copyright 2023 Wiley-VCH GmbH.

Recent progress has further highlighted subnanometer mixed-element architectures as a particularly effective route to accelerate alkaline HOR.^97^ A representative case is the PtRuNiCoFeMo HEA subnanometer nanowires supported on carbon (HEA SNWs/C), which show a rapid rise of anodic current and an early approach to a high, steady HOR current in H_2_-saturated 0.1 M KOH compared with HEA nanoparticles, commercial PtRu/C, and Pt/C (Figure 17A), consistent with faster kinetics in the low-overpotential region as also reflected by the corresponding Tafel behavior (Figure 17B). Quantitatively, at 50 mV vs RHE the HEA SNWs/C achieve a mass activity of 6.75 A mg_Pt + Ru_^–1^ and a specific activity of 8.96 mA cm^–2^, exceeding HEA NPs/C, PtRu/C, and Pt/C by 2.8 and 2.6 times, 4.1 and 2.4 times, and 19.8 and 18.7 times, respectively (Figure 17C). Durability is similarly notable: after 2000 accelerated durability cycles, the mass activity loss of HEA SNWs/C is only 6.2%, whereas HEA NPs/C, PtRu/C, and Pt/C show substantially larger decays of 20.6, 31.5, and 35.3% (Figure 17D). The nanowire catalyst also demonstrates strong impurity tolerance under CO, where chronoamperometry in 1000 ppm of CO in H_2_ shows only a 26.4% activity decrease after 2000 s, while HEA NPs/C and PtRu/C decay by 61.5 and 82.9%, and Pt/C fully deactivates within the test window (Figure 17E). Mechanistically, DFT calculations connect these performance gains to concurrent optimization of hydrogen and hydroxyl interactions on the heterogeneous HEA surface. Across multiple adsorption motifs, the hydrogen binding energies on the HEA nanowires shift toward a more thermoneutral regime compared with Pt and PtRu reference surfaces (Figure 17F), facilitating efficient H adsorption and desorption turnover, while the calculated hydroxyl binding landscape indicates moderately strong OH affinity on selected metal sites that can promote OH assisted interfacial steps in alkaline media (Figure 17G). Consistent with this picture, the computed reaction energetics for water formation and desorption are strongly favorable on the HEA surface, with an overall exothermicity of 0.22 eV and a low barrier of 0.08 eV, in sharp contrast to the endothermic overall profiles on Pt and PtRu (0.57 and 0.27 eV) (Figure 17H). Collectively, these results illustrate how compositional complexity, subnanometer morphology, and site heterogeneity in mixed noble and non-noble HEAs can co optimize key adsorption energetics and reaction thermodynamics, translating into high activity, improved stability, and enhanced CO tolerance for alkaline HOR.

**Figure 17:**
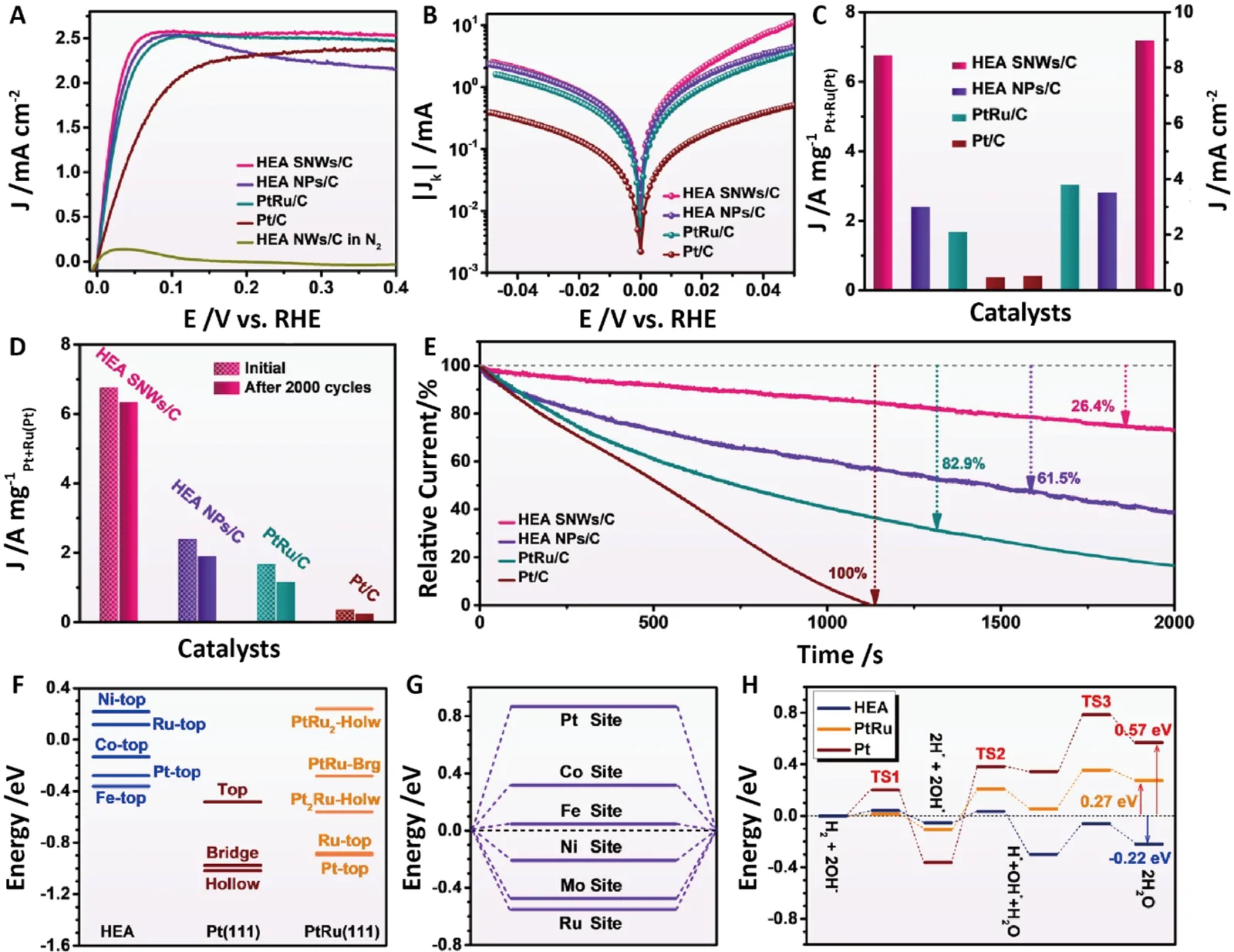
HOR performance, stability, and calculated adsorption energetics of HEA SNWs/C. (A) HOR polarization curves recorded in H_2_-saturated 0.1 M KOH. (B) Corresponding Tafel plots. (C) Mass-normalized activity and ECSA-normalized specific activity evaluated at 50 mV vs RHE. (D) Comparison of mass activity and specific activity measured before and after 2000 ADT cycles. (E) Chronoamperometric current retention for different catalysts in 0.1 M KOH saturated with 1000 ppm of CO/H_2_ at 0.1 V vs RHE. (F) Comparison of hydrogen binding energies (HBE) on HEA SNWs/C versus Pt(111) and PtRu(111), with adsorption at hollow and bridge sites (Holw, hollow; Brg, bridge). (G) Hydroxyl binding energies (OHBE) on HEA SNWs/C across representative metal top sites. (H) Calculated reaction energy profiles for HOR on HEA SNWs/C, Pt(111), and PtRu(111).^97^ Adapted from ref 97 under a Creative Commons Attribution 4.0 International License.

Interestingly, IrRuRhMoW HEA nanoparticles in the sub-2 nm regime provide a clear case where microstrain serves as an explicit design lever to co-optimize activity and stability for alkaline HOR.^100^ Atomic-resolution HAADF-STEM reveals pronounced lattice distortion and irregular atomic arrangements with contrast variations, consistent with multielement mixing in a high-entropy lattice (Figure 18A). The FFT of an individual nanoparticle exhibits overlapping diffraction features (Figure 18B), and crystallographically resolved IFFT images directly visualize lattice dislocations and local bending, where the regions marked as “T” denote dislocations and the red curves highlight distortion trajectories (Figure 18 (C)). Quantitative geometric phase analysis (GPA) further maps the strain field through ε_*xx*_ and ε_*xy*_ components referenced to the (110) and (011) planes (Figure 18D,E): warm colors indicate local expansion and cool colors indicate contraction. The spatially heterogeneous strain distribution confirms that compressive and tensile microstrain coexist within the same nanoparticle rather than appearing as a uniform average distortion. Consistently, 3D atom-overlapping Gaussian-function fitting of the selected region shows disordered atomic column intensities and a broadened projection profile (Figure 18F), corroborating a distortion-driven microstrain landscape at the subnanometer scale. This microstrain-rich structure correlates with markedly improved HOR kinetics and operational robustness. In H_2_-saturated 0.1 M KOH at 1600 rpm, IrRuRhMoW/C exhibits a much steeper rise in anodic current than IrRuRh/C and Pt/C, indicating faster interfacial kinetics in the low-overpotential regime (Figure 18G). The catalyst reaches a PGM-mass-normalized kinetic current density (*j*_k,m_) of 8.09 mA μg_PGM_^–1^ at 50 mV vs RHE and a high PGM-ECSA-normalized specific activity (*j*_k,s_) of 8.76 mA cm_PGM_^–2^, placing it well above the ternary IrRuRh reference and Pt/C under identical normalization (Figure 18H,I). Electrochemical signatures further support adsorption tuning on the HEA surface: the HUPD feature shifts to lower potential (≈0.18 V), consistent with weakened hydrogen adsorption, while the CO-stripping peak shifts to lower potential (≈0.67 V), implying enhanced surface oxophilicity and greater availability of OH_ad_ to facilitate alkaline interfacial steps (Figure 18J). Importantly, this balanced adsorption landscape translates into stability and impurity tolerance: in 100 ppm of CO/H_2_ the current decays only modestly during polarized operation compared with Pt/C (Figure 18K).

**Figure 18:**
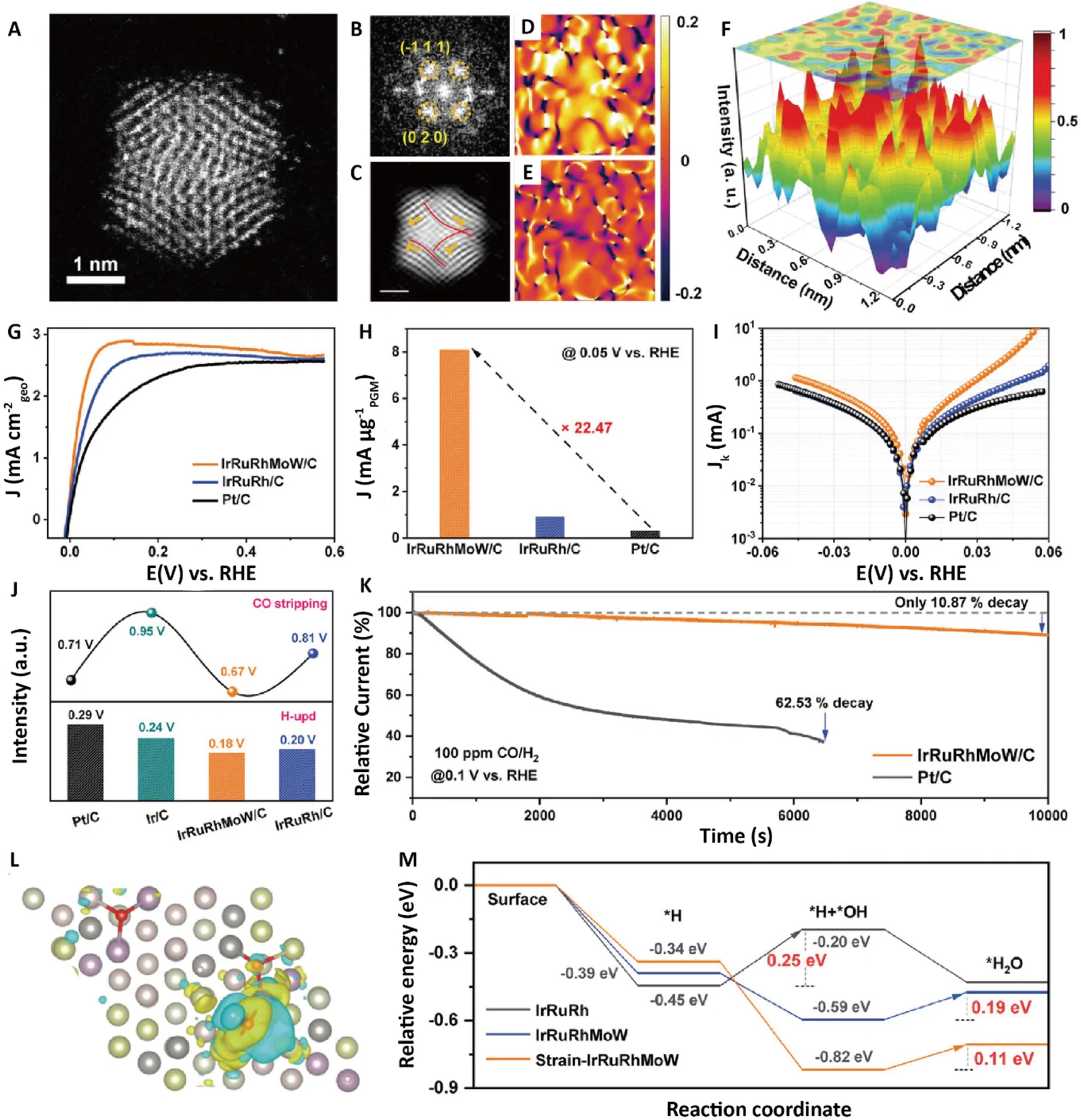
Atomic-scale structure, HOR performance, and reaction energetics. (A) STEM image of an IrRuRhMoW nanoparticle. (B) Corresponding FFT pattern. (C) Inverse FFT (IFFT) image of the (−1 1 1) and (0 2 0) planes, showing abundant dislocations marked by “T” and red lines. (D, E) Strain distributions in the e_*xx*_ and e_*xy*_ directions derived from geometric phase imaging. (F) Three-dimensional topographic image showing atomic-site intensity variations and the relative intensity distribution. (G) HOR polarization curves of IrRuRhMoW/C, IrRuRh/C, and Pt/C. (H) Mass activities measured at 0.05 V vs RHE. (I) Tafel plots. (J) Comparison of CO stripping and H_upd_ peaks. (K) Chronoamperometric current retention in 0.1 M KOH saturated with 100 ppm of CO/H_2_ at 0.1 V vs RHE. (L) Charge density difference of the adsorption configuration. (M) Calculated reaction energies of HOR on IrRuRh, IrRuRhMoW, and strain IrRuRhMoW.^100^ Adapted with permission from ref 100. Copyright 2024 Wiley-VCH GmbH.

DFT calculations provide a consistent mechanistic picture for why microstrain is beneficial in this HEA system. By comparing IrRuRh, IrRuRhMoW, and microstrained IrRuRhMoW models, the calculations show that incorporating oxophilic Mo and W and inducing microstrain collectively modulate the local electronic structure of Ir, Ru, and Rh, including a downshift of the d-band center together with reduced metal-H bonding state occupancy to 0.11 eV, which is consistent with weakened H adsorption. In parallel, charge-density difference analysis reveals intensified electron transfer between strain-IrRuRhMoW and OH, with enhanced charge accumulation toward the OH intermediate, supporting strengthened OH adsorption on the microstrained surface (Figure 18L). Consistent with these descriptors, the calculated free-energy diagram shows that strain-IrRuRhMoW achieves a weakened Δ*E*_H_ of −0.34 eV and an enhanced Δ*E*_OH_ of −0.82 eV, thereby lowering the potential-determining barrier to 0.11 eV, compared with 0.19 eV for IrRuRhMoW and 0.25 eV for IrRuRh (Figure 18M). Collectively, experimental characterization and theory converge on the same design principle: ligand effects from multielement mixing, amplified by microstrain, simultaneously weaken H_ad_ and enhance OH_ad_, co-optimizing the adsorption landscape and lowering the key interfacial barrier, which ultimately secures high alkaline HOR activity together with sustained stability.

The bimetallic and multimetallic case studies in this section collectively highlight that “synergy” in alkaline HOR is most robust when it is intentionally engineered as complementary motifs on the same catalyst, rather than pursued as a single averaged descriptor. In practice, the most effective compositions separate the requirements of hydrogen activation and hydroxyl-related interfacial chemistry into distinct but proximal site types, so that H* generation, OH^–^assisted site regeneration, and water formation can proceed cooperatively with minimal adsorption competition. This perspective also clarifies how to choose elements: start from a PGM-rich backbone to secure fast H_2_ activation and corrosion resistance, then introduce a second or third component only if it provides a clear, mechanistically motivated function such as moderate oxophilicity to increase OH_ad_ availability, a ligand effect that shifts H binding toward the near-thermoneutral window, or a strain or defect lever that broadens the adsorption-strength distribution in a beneficial way. The design target is therefore not “stronger OH adsorption” or “weaker H adsorption” alone, but a balanced adsorption landscape where hydrogen and hydroxyl-related steps can be co-optimized without excessive site blocking, while maintaining resistance to poisoning and potential-driven reconstruction. Taken together, these principles make multimetallic catalysts particularly promising for alkaline HOR, because they offer a uniquely expandable design space in which composition, local ensemble statistics, and strain or defect landscapes can be coengineered to realize functions that are difficult to achieve simultaneously in simpler systems.

### 3.4 Cross-Comparison of Representative Alkaline HOR Catalysts

To facilitate direct comparison across the catalyst examples discussed above, Table 2 summarizes representative alkaline HOR catalysts according to their compositional complexity, including monometallic, bimetallic, trimetallic, tetrametallic, and multimetallic alloy systems. The table compiles key benchmarking metrics, including exchange current density and mass activity, together with the corresponding literature source where available. This side-by-side comparison shows that increasing compositional complexity does not automatically guarantee higher alkaline HOR activity; rather, performance improvements depend on whether the introduced elements generate productive local ensembles, balance HBE and OHBE, regulate interfacial water or oxygenated species, and improve resistance to poisoning or degradation. Therefore, multimetallic design should be guided by mechanistic complementarity among constituent elements rather than by compositional complexity alone. It should be noted that Table 2 is not intended to rank catalysts under identical conditions; instead, it provides a compact comparison of reported activity metrics and highlights the diversity of benchmarking practices across alkaline HOR studies. Cases in which *J*_0,s_ or *J*_m_ metrics were not reported are denoted as “N/A”, further underscoring the need for more standardized benchmarking and reporting across future studies.

**Table 2:** Comparison of Representative HOR Catalysts Discussed in this Review^a^

Catalysts	Metals	Exchange current density (*J*_0,s_, mA cm^–2^)	Mass activity (*J*_m_, A mg^–1^)	ref
Pt	Monometallic alloy	0.16	0.148@50 mV	^87^
Pd@RuIr	Bimetallic alloy	0.47	0.217@50 mV	^87^
IrMo_0.59_	Bimetallic alloy	1.15	0.5@10 mV (*J*_k,m_)	^89^
Ni_4_Mo	Bimetallic alloy	2.82 (*J*_0,g_)	0.078@50 mV (*J*_k,m_)	^94^
i-In_3.9_-PR	Trimetallic alloy	1.86	8.51@50 mV	^95^
Ir_0.2_Mo_0.4_Cu_0.4_	Trimetallic alloy	N/A	0.9@50 mV	^96^
i-ZnIn-PR	Tetrametallic alloy	1.98	10.2@50 mV	^95^
PdNiRuIrRh	Multimetallic alloy	1.19	3.25@50 mV	^98^
PdPtRhIrRu	Multimetallic alloy	N/A	0.214@50 mV	^99^
IrRuRhMoW	Multimetallic alloy	N/A	8.09@50 mV	^100^
PtRuNiCoFeMo	Multimetallic alloy	0.97	6.75@50 mV	^101^

a All HOR data summarized in this table were measured in H_2_-saturated 0.1 M KOH at 1600 rpm.

## 4 Conclusion and Future Perspectives

This Review has surveyed recent progress toward mechanistically informed design rules for bimetallic and multimetallic nanocatalysts that harness synergistic effects to overcome the kinetic limitations of alkaline HOR performance. We first consolidated the current mechanistic picture and its major points of contention by focusing on how HBE, OHBE, and interfacial water can each dominate, or cogovern, alkaline HOR kinetics depending on the operating regime. In particular, we highlighted representative viewpoints that emphasize HBE as a primary descriptor, as well as bifunctional concepts that link activity gains to oxophilic sites and OH related chemistry. We then discussed a rapidly emerging perspective in which interfacial water structure and its coupling to composition and surface oxygenated fragments can reshape hydrogen transfer pathways and provide activity trends that are not readily rationalized by adsorption energetics alone. To connect mechanism to measurable behavior, we summarized experimental approaches that quantify hydrogen adsorption and poisoning tolerance, and operando spectroscopies that probe potential dependent surface states and interfacial species, alongside DFT as a framework for evaluating adsorption energetics, elementary barriers, and inter element synergy within bimetallic and multimetallic ensembles. Building on these mechanistic foundations, we surveyed composition-driven strategies across catalyst families, from bimetallic systems to trimetallic catalysts and HEAs, emphasizing how elemental selection and local coordination can tune ensemble availability, modulate adsorption strengths, reshape the interfacial water environment, and enhance tolerance to poisoning species such as CO. Finally, we noted that stability and durability can also be coupled to interfacial solvent structure and surface oxidation behavior, reinforcing that alkaline HOR performance should be evaluated through the joint lens of activity, poisoning resistance, abvnd long-term structural robustness.

Despite this progress, major gaps remain in achieving a predictive understanding of alkaline HOR and in translating mechanistic insights into catalysts that operate reliably under device-relevant AEMFC conditions. To consolidate the design principles discussed throughout this Review, we highlight six design rules and future directions that we believe will shape the next phase of alkaline HOR research toward practical AEMFC anodes.

(1) **Understanding the impact of site heterogeneity and adsorption distributions in multimetallic catalysts**. For compositionally complex multimetallic surfaces, adsorption should be treated as a distribution rather than a single value. Compared with monometallic and simple bimetallic catalysts, trimetallic systems and multicomponent alloys, including five-component HEAs, intrinsically generate a wide spectrum of surface motifs with distinct local coordination and electronic environments.^101^ Consequently, the binding strengths of key intermediates such as H* and OH* are better described by site-resolved distributions than by an “average” HBE or OHBE. Relying only on mean adsorption energetics can obscure the minority ensembles that dominate turnover or, conversely, the strongly binding motifs that promote site blocking and poisoning. In addition, adsorbates, intermediates, and even products may diffuse, reorganize, and exchange among neighboring motifs under polarization, further complicating mechanistic interpretation. Disentangling these effects will require a closer integration of operando methods such as XAS, that track surface populations and local environments. Complementary theory is equally important. In particular, DFT calculations and related machine-learning approaches can be used to evaluate adsorption energies and elementary barriers on representative ensembles, quantify how local coordination and elemental identity shift HBE and OHBE, and provide a foundation for linking adsorption distributions to kinetically relevant active-site motifs. Importantly, recognizing adsorption as a distribution also reframes multimetallic catalyst design: rather than optimizing a single “best” binding energy, effective strategies should aim to shape the distribution by increasing the fraction of productive ensembles, suppressing strongly binding poisoning motifs, and maintaining cooperative site proximities that support efficient H*–OH* coupling and interfacial water reorganization under operating conditions.
(2) **Interfacial solvation structures as a design variable**. Recent studies make a strong case that alkaline HOR activity can be tuned not only by H* and OH* adsorption energetics, but also by how a catalyst organizes cations and water within the electric double layer. For example, Men and co-workers showed that shifting the potential of zero charge can regulate alkali-metal cation packing and, in turn, the ordering and connectivity of the interfacial hydrogen-bond network, lowering effective proton-coupled charge-transfer barriers and accelerating HOR kinetics.^21^ Complementarily, Wan and co-workers demonstrated that introducing Rh-based hydroxyl motifs can reorganize the Pt surface water structure and markedly boost alkaline HOR, highlighting that local oxygenated fragments can act as “water-structure directors” rather than merely spectators.^20^ In the same spirit, electric-double-layer perspectives of surface oxophilicity emphasize that how hydroxyl is stabilized can reshape hydrogen-bond connectivity and local interfacial fields, thereby changing kinetics even when conventional adsorption descriptors appear similar.^19^ These insights translate into practical design levers: selecting compositions and surface motifs that tune potential of zero charge and moderate oxophilicity, engineering bifunctional ensembles that template a connected water network while avoiding excessive oxygenated coverage, and pairing catalyst surfaces with electrolytes or ionomers whose cation environment reinforces the desired solvation structure. In practice, establishing causal links between interfacial solvation structures and alkaline HOR kinetics still relies heavily on theory. While operando spectroscopy can provide valuable fingerprints of surface states and adsorbate populations, it remains challenging to directly and quantitatively resolve the potential-dependent configurations of water, cations, and hydrogen-bond networks within the electric double layer under realistic reaction conditions. As a result, computational approaches, ranging from solvation-corrected DFT and constant-potential methods to explicit-solvent simulations and microkinetic modeling, are often required to bridge experimental trends to molecular-level descriptors, such as cation proximity, interfacial field strength, hydrogen-bond connectivity, and the associated changes in elementary barriers.
(3) **Developing HEA catalyst design strategies**. HEA catalysts offer an appealing platform for meeting the demanding requirements of alkaline HOR, yet their compositional complexity makes it challenging to define reliable, generalizable design strategies. One practical starting point is a Sabatier-principle-guided approach, in which the goal is to maintain a balanced window of hydrogen- and hydroxyl-related interactions while avoiding excessive stabilization of oxygenated species that can block active ensembles. Based on typical volcano plots, PGMs such as Ir, Pt, and Ru, often exhibit relatively suitable affinities for both H* and OH*, but their cost motivates incorporating nonprecious elements without sacrificing the adsorption balance. An actionable design concept is therefore elemental pairing by compensation: combining elements with stronger H* and OH* binding tendencies (e.g., Mo, W, Fe, Co, and Ni) with elements that bind more weakly (e.g., Cu, Al, and Bi) to tune the ensemble landscape toward an optimal range of HBE and OHBE.^96,101^ Beyond activity, introducing nonprecious components may also reshape poisoning pathways, potentially improving CO tolerance by modifying ensemble availability and the distribution of adsorption strengths. Moving forward, these considerations, activity, adsorption balance, poisoning resistance, and durability, should be evaluated together, underscoring both the opportunity and the complexity inherent to HEA catalyst design for practical alkaline HOR.
(4) **From composition screening to facet-guided catalyst design**. Recent years have seen the discovery of many alloy compositions with outstanding alkaline HOR performance, including IrRu, PtRu, and a range of multimetallic formulations. However, in most reported systems, catalysts are predominantly spherical or structurally irregular, meaning that their surfaces lack well-defined crystallographic facets and do not present a controlled atomic arrangement. This limitation is nontrivial because surface structure, specifically the symmetry of atomic arrangements and the distribution of site motifs, can directly govern both HOR activity and stability. Moving beyond composition-only screening to facet-guided catalyst design, and identifying optimal composition–facet combinations, is therefore essential for achieving both higher activity and improved durability. In this context, our recent findings provide a representative example: an IrRu bimetallic surface enclosed by FCC facets with a square atomic arrangement delivers markedly improved activity and durability compared with commercial Pt/C.^87^ More broadly, the vast compositional and structural diversity of HEA catalysts offers an exceptional platform for tailoring catalytic performance. By combining multiple metallic elements in tunable ratios and placing them on distinct crystallographic facets, it becomes possible to access an immense design space for optimizing active-site motifs, adsorption energetics, and reaction pathways, while simultaneously addressing stability and poisoning resistance under alkaline HOR conditions. Looking forward, epitaxial growth provides a practical route to facet control by enabling layer-by-layer construction of multimetallic surfaces with targeted crystallographic orientations and atomic arrangements. Systematic electrochemical HOR benchmarking on such facet-defined catalysts, coupled with theory-driven screening, should accelerate the identification of composition and facet pairs that deliver optimal intermediate binding, favorable reaction pathways, and robust stability under alkaline operation.
(5) **Stability and passivation challenges for catalysts containing nonprecious metals under start–stop conditions**. Incorporating nonprecious elements is a practical route to reduce PGM loading, but it can introduce stability penalties that strongly influence alkaline HOR performance.^7,8^ A central issue is passivation: nonprecious components may form surface (oxy)­hydroxides under anodic potentials or potential excursions, which block hydrogen-active ensembles and reshape the interfacial reaction environment.^55^ Ni-containing catalysts provide a representative example, where oxidation to Ni­(OH)_2_ or NiOOH at elevated potentials can suppress HOR activity and accelerate surface reconstruction.^58,94^ These challenges become more severe under start–stop and fuel-starvation events. During such transients, H_2_/air fronts can create oxidizing conditions with interfacial potentials exceeding ∼0.8 V vs RHE, thereby accelerating oxidation, passivation, and corrosion processes.^102–105^ Addressing this issue calls for durability-aware design that goes beyond tuning HBE and OHBE, including alloy environments that suppress deep oxidation, surfaces that remain permeable to H_2_ activation, and stress tests that explicitly emulate start–stop operation.^45,94^ A complementary strategy is to pair nonprecious components with suitable PGMs to improve oxidation resistance while maintaining activity, for example, Pt–Ni and related PGM–M alloys can, in some cases, mitigate irreversible oxidation of the nonprecious component through electronic and ensemble effects. Operando diagnostics (e.g., Raman and XAS) combined with theory will be essential to track oxidation pathways, identify failure modes, and establish stability-oriented design rules for practical AEMFC anodes.^34,37^

(6) **Validate catalysts under device-level and manufacturable conditions**. Although many bimetallic and multimetallic catalysts exhibit promising alkaline HOR activity in RDE measurements, their practical relevance must be further validated under device-level AEMFC conditions, such as flow-cell or single-cell MEA configurations.^3,106^ In practical electrodes, catalyst performance can be strongly affected by ionomer adsorption and distribution, catalyst–ionomer interactions, local water management, gas transport, catalyst-layer architecture, carbonate contamination, and three-phase-boundary formation.^3,106,107^ These factors may alter accessible active sites, interfacial water structure, hydroxide transport, and apparent durability, especially under high current densities and high ionomer coverage.^105,107^ Therefore, future studies should move beyond intrinsic activity metrics and evaluate catalysts under realistic operating and stress conditions, including high-current-density operation, long-term galvanostatic or potentiostatic holds, start–stop cycling, fuel starvation, CO- or CO_2_-containing feed streams, and accelerated stress tests. When available, MEA-level activity and durability should be compared with RDE-derived trends to determine whether HBE-based, bifunctional, or interfacial water design principles remain valid in practical catalyst layers.^105,107^

From a manufacturing perspective, scalable catalyst production and electrode fabrication should be considered together with intrinsic activity and durability. For catalyst production, continuous-flow droplet reactors and microreactors are promising routes for scalable nanoparticle synthesis because they provide better control over precursor mixing, residence time, heat and mass transfer, feeding rate, and reaction atmosphere.^108–111^ These features are important for maintaining compositional reproducibility, particle-size uniformity, alloying degree, and phase homogeneity in multimetallic catalysts. Another scalable route is high-temperature synthesis, including impregnation–reduction, thermal annealing, and carbothermal reduction on conductive supports.^96,112–114^ These methods are compatible with larger-scale powder production and can directly generate supported alloy catalysts, although particle sintering, elemental segregation, incomplete alloying, and surface oxidation/passivation must be carefully controlled.

Beyond catalyst powder production, scalable electrode-coating methods are also required to translate HOR catalysts into large-area AEMFC electrodes. In practical MEA fabrication, supported catalysts must be formulated into stable catalyst inks and uniformly deposited by coating techniques such as spray coating, blade coating, roll-to-roll coating, or slot-die coating.^3,106,115–117^ Ink formulation and coating conditions directly affect catalyst-layer porosity, ionomer distribution, gas transport, water management, ionic conduction, and the accessibility of HOR active sites.^107^ Therefore, catalyst manufacturability should be evaluated not only at the powder level, but also through coating quality, electrode uniformity, catalyst-layer adhesion, and MEA performance reproducibility.^105^ Standardized reporting of catalyst loading, ECSA normalization, mass-transport correction, ionomer content, operating current density, degradation rate, and electrode-fabrication parameters will be essential for distinguishing intrinsic catalytic improvements from electrode- or device-dependent effects.

Looking forward, advancing alkaline HOR from mechanistic insight to practical AEMFC anodes will require a tighter coupling between materials design, interface engineering, and device-relevant validation. Progress will be accelerated by workflows that integrate controllable synthesis of well-defined multimetallic surfaces with *operando* characterization capable of resolving potential-dependent adsorbate populations and interfacial solvation structures, alongside theory and data-driven screening that can navigate the vast composition–structure space of multimetallic catalysts. Equally important is to evaluate catalysts under realistic stressors, including electrolyte environments, water-management constraints, and start–stop transients, so that activity gains are not achieved at the expense of passivation, corrosion, or surface reconstruction. Ultimately, establishing design rules that remain predictive across compositions, facets, and operating conditions should enable alkaline HOR electrocatalysts that combine high intrinsic activity with the durability required for long-term AEMFC deployment.
